# All-cause, cardiovascular disease and cancer mortality in the population of a large Italian area contaminated by perfluoroalkyl and polyfluoroalkyl substances (1980–2018)

**DOI:** 10.1186/s12940-024-01074-2

**Published:** 2024-04-16

**Authors:** Annibale Biggeri, Giorgia Stoppa, Laura Facciolo, Giuliano Fin, Silvia Mancini, Valerio Manno, Giada Minelli, Federica Zamagni, Michela Zamboni, Dolores Catelan, Lauro Bucchi

**Affiliations:** 1 Unit of Biostatistics, Epidemiology and Public Health, Department of Cardiac, Thoracic, Vascular Sciences and Public Health University of Padua, Padua, Italy; 2 Comitato mamme NO-PFAS, Vicenza, Padua, Verona Italy; 3 Emilia-Romagna Cancer Registry, Romagna Cancer Institute, IRCCS Istituto Romagnolo per lo Studio dei Tumori (IRST) Dino Amadori, Meldola, Forlì, Italy; 4 Statistical Service, Istituto Superiore di Sanità, Rome, Italy

**Keywords:** Perfluoroalkyl substances, Polyfluoroalkyl substances, PFAS, PFOA, PFOS, Cardiovascular disease, Kidney cancer, Testicular cancer

## Abstract

**Background:**

Per- and polyfluoroalkyl substances (PFAS) are associated with many adverse health conditions. Among the main effects is carcinogenicity in humans, which deserves to be further clarified. An evident association has been reported for kidney cancer and testicular cancer. In 2013, a large episode of surface, ground and drinking water contamination with PFAS was uncovered in three provinces of the Veneto Region (northern Italy) involving 30 municipalities and a population of about 150,000. We report on the temporal evolution of all-cause mortality and selected cause-specific mortality by calendar period and birth cohort in the local population between 1980 and 2018.

**Methods:**

The Italian National Institute of Health pre-processed and made available anonymous data from the Italian National Institute of Statistics death certificate archives for residents of the provinces of Vicenza, Padua and Verona (males, *n* = 29,629; females, *n* = 29,518) who died between 1980 and 2018. Calendar period analysis was done by calculating standardised mortality ratios using the total population of the three provinces in the same calendar period as reference. The birth cohort analysis was performed using 20–84 years cumulative standardised mortality ratios. Exposure was defined as being resident in one of the 30 municipalities of the *Red area*, where the aqueduct supplying drinking water was fed by the contaminated groundwater.

**Results:**

During the 34 years between 1985 (assumed as beginning date of water contamination) and 2018 (last year of availability of cause-specific mortality data), in the resident population of the *Red area* we observed 51,621 deaths vs. 47,731 expected (age- and sex-SMR: 108; 90% CI: 107–109). We found evidence of raised mortality from cardiovascular disease (in particular, heart diseases and ischemic heart disease) and malignant neoplastic diseases, including kidney cancer and testicular cancer.

**Conclusions:**

For the first time, an association of PFAS exposure with mortality from cardiovascular disease was formally demonstrated. The evidence regarding kidney cancer and testicular cancer is consistent with previously reported data.

**Supplementary Information:**

The online version contains supplementary material available at 10.1186/s12940-024-01074-2.

## Background

The synthetically-made industrial chemicals per- and polyfluoroalkyl substances (PFAS) have been marketed since the 1940s and have been extensively used since the 1950s in a wide variety of applications [1]. According to the U.S. National Institute of Environmental Health Sciences, there are some 15,000 different PFAS molecules, but hundreds of newer and previously unreported PFAS and PFAS-related compounds –the so-called “emerging” PFAS– continue to be identified by researchers [2, 3].

PFAS have the unique property of a hydrophobic-lipophilic carbon chain with fluorine atoms instead of hydrogen atoms in at least part of the carbon atoms [4]. Since the carbon-fluorine bond is among the strongest ones, PFAS have a high resistance to degradation. This makes them industrially valuable, especially in applications at high temperatures or high pressures or in corrosive environments [1–3].

Closely linked to this industrial success, however, there is the problem that PFAS are difficult to break down through normal chemical, physical or biological processes, including conventional water/wastewater treatment processes [5]. This causes their persistence in the soil, water, air and organisms [6]. PFAS have been found all over the world in a variety of environmental media [5], including surface, ground and drinking water [4, 7, 8]. Quantitatively, manufacturing facilities are the most important sources of contamination [9, 10] followed by emissions from municipal and industrial wastewater treatment plants [9, 11–13].

People are most commonly exposed to PFAS by consuming contaminated water or food (including contamination from food packing), or using products made with PFAS, or breathing PFAS-contaminated air. Given their persistence, the serum levels of some PFAS may increase over long periods of time [14]. Increasing epidemiologic research has suggested that exposure to high levels of PFAS might be associated with many adverse health conditions, including decreased fertility in men [15] and women [14], birth defects [16], delayed development [17], osteoporosis in young people [18, 19], damage to the immune system [20], reduced antibody response after vaccination, allergy and asthma in children [21], liver disease [22], increased serum levels of cholesterol [23], impaired thyroid function [24], insulin resistance [25], gestational diabetes [26], pregnancy-induced hypertension [27], neuro-endocrine disruption [28] and cancer.

With respect to the latter, the International Agency for Research on Cancer (IARC) has re-evaluated the carcinogenicity of perfluorooctanoic acid (PFOA) and perfluorooctanesulfonic acid (PFOS) in November 2023 [29]. PFOA has been classified as ‘carcinogenic to humans’ (Group 1) based on ‘sufficient’ evidence in experimental animals and ‘strong’ mechanistic evidence in exposed humans, indicating an association with epigenetic alterations and an immunosuppressive action. Additionally, there is ‘limited’ epidemiologic evidence in humans for an association between PFOA and the risk of kidney cancer and testicular cancer. PFOS has been classified as ‘possibly carcinogenic to humans’ (Group 2B) based on ‘strong’ mechanistic evidence. The evidence for an association between PFOS exposure and cancer in experimental animals has been considered to be ‘limited’ and the evidence regarding cancer in humans ‘inadequate’. This is due to the fact that the few available studies have led to positive findings only sporadically and inconsistently. It appears that there remains much room for further epidemiologic research.

An ever-increasing number of contaminated sites are being discovered in many countries [30]. An important evidence regarding PFAS exposure has come from the C8 Health Project, a series of population studies conducted to investigate the community living near the DuPont Washington Works fluoroproduct manufacturing facility in Parkersburg, West Virginia, U.S. [31–34]. However, the world’s largest episode of PFAS water contamination reported so far occurred in Italy [35]. In 2011, the Institute of Water Research of the National Research Council (IRSA–CNR) was commissioned a study by the Italian Ministry for Environment, Land and Sea Protection to investigate the contamination with PFAS in the most important river basins of the Country [36]. In 2013, the study results revealed the surface, ground and drinking water contamination of part of the provinces of Vicenza, Padua and Verona, situated in the Veneto Region (northern Italy), in which about 125,000 persons were involved according to early estimates [13, 35, 37].

In the present article, we report a study on the temporal evolution of all-cause mortality and selected cause-specific mortality by calendar period and birth cohort in the population living in the contaminated area between 1980 and 2018.

## Methods

### Study setting: environmental monitoring and source, area and timing of contamination

#### Environmental monitoring plan

The results of the study on the contamination with PFAS in the Italian river basins [37] were communicated to the Veneto Regional Administration on 11 July 2013 [38]. Soon after, the Environmental Prevention and Protection Agency of the Veneto Region (ARPAV) established an environmental monitoring plan, which is still operating, with the two-fold objectives of (1) assessing the geographic extension and the level of groundwater and drinking water contamination with PFAS and (2) identifying its source(s). Originally, 12 types of PFAS were being measured in water matrices, namely: perfluorobutanoic acid (PFBA), perfluorobutanesulfonic acid (PFBS), perfluoropentanoic acid (PFPeA), perfluorohexanoic acid (PFHxA), perfluorohexanesulfonic acid (PFHxS), perfluoroheptanoic acid (PFHpA), PFOA, PFOS, perfluorononanoic acid (PFNA), perfluorodecanoic acid (PFDeA), perfluoroundecanoic acid (PFUnA), and perfluorododecanoic acid (PFDoA) [35].

Chemical analysis of drinking water samples during 2013 indicated the presence of PFOA (median, 319 ng/L), PFBA (123 ng/L), PFBS (91 ng/L), PFPeA (70 ng/L), PFHxA (52 ng/L), PFOS (18 ng/L), PFHpA (14 ng/L) and PFHxS (< 10 ng/L) [35]. Further details can be found in another publication [39].

#### Source of contamination with PFAS

The Miteni (formerly Ri.Mar) factory, a manufacturing plant located in the town of Trissino (province of Vicenza), was identified as the most likely source of contamination with PFAS in the Veneto Region. The factory was established in the 1950s. The production of PFAS began in 1966 and was terminated in 2018 [40]. Long-chain PFAS (containing ≥6 carbons, such as the PFOA and the PFOS) were produced between 1968 and 2013 and short-chain PFAS (for example, the PFBA) between 2001 and 2016 [40, 41].

The Miteni factory discharged the treated effluent into the municipal wastewater treatment plant, the output of which was mixed with the outputs of other four plants and carried by a single sewer pipeline (known as *collettore Arica*) to the Fratta-Gorzone river. These five plants collect waters from domestic sewage and industrial districts of the province of Vicenza. This makes it impossible to distinguish the effluent originating from the fluorochemical plant from those discharged by the other industrial activities (mainly textile and tannery factories). However, a comparison between the PFAS composition of the primary discharge of the fluorochemical plant and that of the output of the *collettore Arica* revealed a close similarity [13, 37, 39].

#### Area of water contamination with PFAS

The contamination of surface water and groundwater took place in the area located downstream of the Miteni factory, which has a well-developed agricultural and food industry. The groundwater contamination plume occupies a surface area of 190 km^2^ and involves public aqueducts and private wells across the provinces of Vicenza, Padua and Verona. Based on the results of the environmental monitoring, the ARPAV identified 30 municipalities forming an area of maximum exposure (*Red area*) [42], for a total population of 153,525 inhabitants on 1 January 2020 [43], a larger number than previously estimated [13, 35]. The *Red area* is further divided into *Red area A*, including the municipalities that are served by the contaminated aqueduct and are also located on the groundwater contamination plume, and *Red area B*, including municipalities served by the contaminated aqueduct but not located on the groundwater contamination plume.

**Fig. 1:**
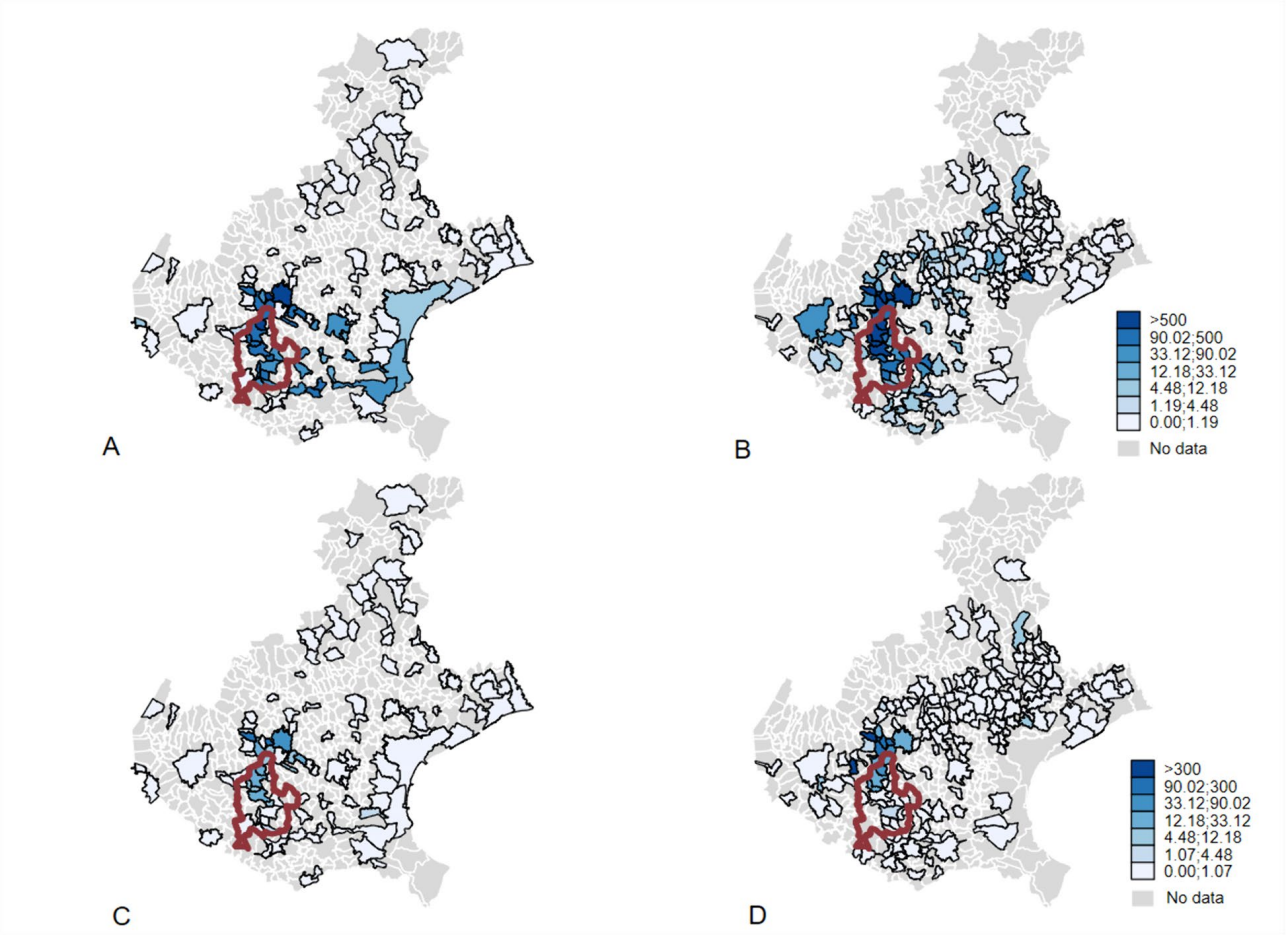
Map of concentrations (ng/L) of perfluorooctanoic acid (PFOA) (top panels **A** and **B**) and perfluorooctanesulfonic acid (PFOS) (bottom panels **C** and **D**) in the surface water (left panels **A** and **C**) and groundwater (right panels **B** and **D**) of the municipalities of the Veneto Region (northern Italy). A red outline indicates the *Red area*. July 2013–June 2015. Source: Environmental Prevention and Protection Agency of the Veneto Region (ARPAV) (https://www.arpa.veneto.it/dati-ambientali/open-data/idrosfera/concentrazione-di-sostanze-perfluoroalchiliche-pfas-nelle-acque-prelevate-da-arpav. Accessed 14 Feb 2024)

The contamination was mapped by the Veneto Regional Administration in 2013. The maps are available online [44]. A digital navigable map is available from another website [45]. Figure 1 shows the map of water contamination by PFOA and PFOS in the years 2013–2015, i.e., when the contamination was discovered and before the full implementation of mitigating and remediation measures. In the map, the 30 municipalities of the *Red area* that are served by the aqueduct carrying contaminated groundwater since 1985 are also shown.

In this study, we defined the exposed population as the resident population of the *Red Area.* The *Red area A* includes the municipalities of (in parentheses, the province to which the municipality belongs) Alonte (Vicenza, VI), Asigliano Veneto (VI), Brendola (VI), Lonigo (VI), Sarego (VI), Noventa Vicentina (VI), Orgiano (VI), Pojana Maggiore (VI), Montagnana (Padua, PD), Cologna Veneta (Verona, VR), Pressana (VR), Roveredo di Guà (VR), and Zimella (VR). The municipalities situated in the *Red area B* are Urbana (PD), Albaredo d’Adige (VR), Arcole (VR), Bevilacqua (VR), Bonavigo (VR), Boschi Sant’Anna (VR), Legnago (VR), Minerbe (VR), Terrazzo (VR), and Veronella (VR). The *Red area B* also includes parts of the municipalities of Agugliaro (VI), Val Liona (VI), Borgo Veneto (PD), Casale di Scodosia (PD), Lozzo Atestino (PD), Megliadino San Vitale (PD), and Merlara (PD).

#### Timing of water contamination with PFAS and mitigating and remediation measures

According to estimates from the ARPAV, the contamination of the groundwater began in 1966 in Trissino [46] and reached Montecchio Maggiore in 1970, Almisano in 1984 and Lonigo –situated further south– in 1985.

In the period 1985–1989, unfortunately, several inter-municipal and municipal aqueducts drew water from the Almisano groundwater source. In addition, they were connected to form a larger public aqueduct with new reservoirs (*Centrale Madonna di Lonigo*), which delivers drinking water to the 30 municipalities in the *Red area*. This aqueduct reached its maximum operational capacity in 1995 [46].

PFAS exposure via drinking water ingestion from the aqueduct serving the *Red area* has been reduced since 2013, with the implementation of a filtration system with granular activated carbon. The levels of specific PFAS were found to be below the threshold of detectability of 5 ng/L in 2018. In the last few years, a new aqueduct has been built that takes water from a northern area of the Veneto Region. This has allowed to close the Almisano groundwater source. The new water delivery system has entered into operation on 8 March 2024.

In the *Red area A*, a small proportion of people were not connected to the aqueduct and obtained their drinking water from private wells (5% on average, with only three municipalities reaching a level varying between 12 and 20%). These wells were not equipped with filtration systems and were heavily contaminated, because PFAS were discharged into the groundwater between 1979 (northernmost municipalities) and 1991 (municipalities situated further south) [41]. Private wells and other aqueducts served approximately 20% of residents in *Red area B* but were not contaminated [42, 47]. As a consequence, getting drinking water from private wells caused a misclassification of exposure in the *Red area B* but not in the *Red area A.*

#### Food contamination with PFAS

In 2016, the Veneto Regional Administration developed a plan for the monitoring of 12 PFAS congeners in local food matrices. After the implementation, the Italian National Institute of Health (ISS) made a scenario-based estimate of the weekly dietary exposure in the *Red area* showing a not negligible contribution to human exposure from contaminated local food consumption [48–50]. In 2021, food contamination and food contribution to total human exposure were assessed using the individual records of the food monitoring campaigns of the years 2016–2017. The analysis showed that food contamination with PFAS was widespread over the entire *Red area* with modest differences [51]. The exportation of local food may have caused exposure outside the *Red area*.

### Health surveillance plan

Between July 2015 and April 2016, the ISS performed the first serum PFAS concentration measurements campaign in the local population [52]. PFOA had the highest concentration but detectable levels of eight other molecules (PFBA, PFBS, PFPeA, PFHxA, PFHxS, PFHpA, PFDoA, and PFOS) were found. The median serum level of PFOA was 14 ng/mL (95th percentile: 248 ng/mL). For PFBA, the median concentration was below the level of detectability and the 95th percentile was 0.6 ng/mL, reflecting its more rapid excretion.

In 2017, the Veneto Regional Administration developed a health surveillance plan for the exposed population [35, 53]. The implementation started in 2017–2018 from the cohorts born in 1962–2002 and was subsequently extended to include the other birth cohorts. The plan is based on active personal invitations to undergo measurements of PFAS in serum, laboratory tests and medical examinations, mainly to assess serum lipids as well as liver and kidney functionality, followed by specialist diagnostic work-up, if necessary. The plan covers the 30 municipalities of the *Red area*.

In Supplementary Tables S1 and S2, the mean, the median and the 95th percentile of serum levels of PFOA and PFOS by attained age at first invitation (range age class: 7–72 years) and year of invitation (2017–2023) to the health surveillance plan are shown [53].

### Demographics, migration balance and deprivation index

The background demographic figures of the reference provinces of Vicenza, Padua and Verona showed a smooth increase between 1981 and 1991, a steeper growth since 1991 and a plateauing since 2011 (Supplementary Fig. S1). There was no appreciable difference between the three provinces and the municipalities of the *Red area*. Peculiar patterns were observed only in the municipalities of Lonigo, where a marked increase occurred between 1981 and 2011, and Montagnana, which showed a decreasing trend since 1981.

The natural balance of the population and the net migration in the years 2003–2017 were consistent with the above patterns, with Lonigo showing a positive balance and Montagnana a negative balance (Supplementary Fig. S2 and S3). The three province capital cities of Vicenza, Padua and Verona exhibited a strong negative trend, with a positive net migration occurring in Verona and Padua.

As shown in the age-sex pyramids (Supplementary Fig. S4), the age distribution in 1982 and 2018 did not differ between the three provinces and the 30 municipalities of the *Red area*. In the latter, it appears that the maximum impact of the PFAS contamination is experienced by the cohorts born between 1960 and 1970, who were aged 10–20 years in 1982.

The socio-economic characteristics of the municipalities of the *Red area* did not differ much from the reference population of the three provinces. In 2011, the material deprivation index was similar (Supplementary Fig. S5). The analysis of the five socio-economic characteristics pertaining to deprivation (low level of education, unemployment, rental housing, single parenting and house crowding) showed only a relatively larger percentage of low-educated people (32% vs. 30%) in the *Red area* (Table 1). A more detailed picture can be found in the biplot reported in Supplementary Fig. S6.

**Table 1:** Percentage of people with low level of education, unemployment, rental housing and single parenting and house crowding index in the provinces of Vicenza, Padua and Verona (northern Italy) as a whole and in the 30 municipalities of the perfluoroalkyl and polyfluoroalkyl substances (PFAS)-contaminated *Red area*. 2011 census

	Low level of education (%)	Unemployement (%)	Rental housing (%)	Single parenting (%)	House crowding index
**Three provinces**	30.41	5.90	11.17	9.59	2.21
***Red area***	32.63	6.14	11.21	9.97	2.07

The *Red area* includes the municipalities connected to the contaminated aqueduct. Low level of education is expressed as a percentage of people with elementary school education or less out of the total population aged 6 years or more. Unemployment is expressed as a percentage of the total labor force. Rental housing is expressed as a percentage of the total occupied housing units. Single parenting is expressed as the percentage of mothers or fathers who live with a child or children and no husband, wife, or partner out of the total families. House crowding index is expressed as the number of people per 100 square meters in occupied houses. Source: Osservatorio Nazionale sulla Salute nelle Regioni Italiane. Rapporto Osservasalute 2009 (https://osservatoriosullasalute.it/osservasalute/rapporto-osservasalute-2009. Accessed 14 Feb 2024)

### Study rationale and design

The rationale of this study is based on the consideration that PFAS contamination in the Veneto Region is extremely important in terms of size of the exposed population and magnitude of exposure. The accident can be taken as a great and tragic *natural* experiment [54]. PFAS exposure of the population began in the second half of the 1980s and depended on the use of contaminated drinking water. The experimental condition consisted in being connected to the aqueduct, which acted as an instrumental variable. The actual PFAS exposure could depend on several factors and, among these, on the determinants of contaminated water consumption for the common uses, i.e., human consumption, cooking and washing while others –like agricultural irrigation– are discouraged or prohibited by the municipal Administrations. The relationship between being connected to the aqueduct and a disease outcome was not affected by confounding, while the relationship between actual PFAS exposure and a disease outcome could be confounded. An instrumental variable should be unrelated to the outcome, strongly related to the exposure and unrelated to the potential confounders. Being connected to the aqueduct was strongly associated with the exposure and was independent from potential confounders and, of course, from the outcome. As a consequence, comparing the population connected to the aqueduct with an unconnected reference population enables to test a potential causal association. Of course, we considered the possibility of residual confounding in the definition of the two populations and of potential misclassification, because we had only information at the municipality level. On average, more than 70% of the population of the *Red area* was served by the aqueduct. In the *Red area A,* furthermore, the population not served by the aqueduct was supplied by private wells containing the contaminated groundwater.

The potential fallacy associated with the use of the information on PFAS exposure at the population level for the definition of the groups being compared can be exemplified by considering Lonigo. This municipality has shown a high serum PFAS concentration in the health surveillance plan and has some specific socio-economic characteristics that are strongly linked to the volume of water consumption [55, 56] and cardiovascular mortality [57] like education and rental housing. This causes socio-economic factors to act as confounders of the association between PFAS exposure and cardiovascular disease (Supplementary Fig. S7).

The availability of mortality data for a time span as long as 1980–2018 enabled a temporal analysis of mortality risk by two different time axes. The temporal variations of mortality were evaluated both by focusing on the effect of the calendar period and by investigating the birth cohort influences. The effect of the calendar period is interpretable as the effect of the total dose of PFAS since 1985. Therefore, mortality in the 1980–1984 calendar period can be assumed as an internal reference, with the subsequent calendar periods being characterised by a mortality experience that reflects growing exposure doses. The effect of the birth cohort, conversely, is interpretable as the effect of the exposure which begins at different ages. The exposure started after the age of (at least) 60 years for the cohort born in 1920–1929 and after the age of 25 years for the cohort born in 1960–1969.

The bioaccumulative nature of PFAS led to a constantly increasing exposure of the population. Since water filtration was implemented starting with 2013, it could potentially impact the last time segment, i.e., 2015–2018. Public health measures, coupled with the health surveillance activities and changes in lifestyle and health behaviour after the discovery of water contamination, may have contributed to a reduction of PFAS exposure of the population.

We used the population of the three provinces where the *Red area* is situated as reference population. In a sensitivity analysis, we used as reference the population of the three provinces excluding the 30 municipalities of the *Red area.* Notice that we also excluded from the exposed population those municipalities with groundwater contamination since 1966 (the so called *Orange area* [47]).

### Mortality data

The ISS pre-processed and made available to us anonymous data from the Italian National Institute of Statistics (ISTAT) death certificate archives for all males (*n* = 29,629) and females (*n* = 29,518) who were residents of the provinces of Vicenza, Padua and Verona and died between 1980 and 2018 (more recent data were not yet ready for use at the time of analysis). Records were extracted by the Statistical Service of the ISS from the ISS cause-specific mortality database using the codes of the International Classification of Diseases (ICD)-9 [58] for the years 1995–2002 and ICD-10 [59] for subsequent years. The ICD-9 codes were converted into ICD-10 codes (Supplementary Table S3). It must be considered that the ICD-10 Chapter IX (I00-I99) contains some codes indicating diseases not related to PFAS according to the current literature. Our definition of circulatory disease is based on the ICD-10 Chapter IX codes.

### Statistical analysis

**Fig. 2:**
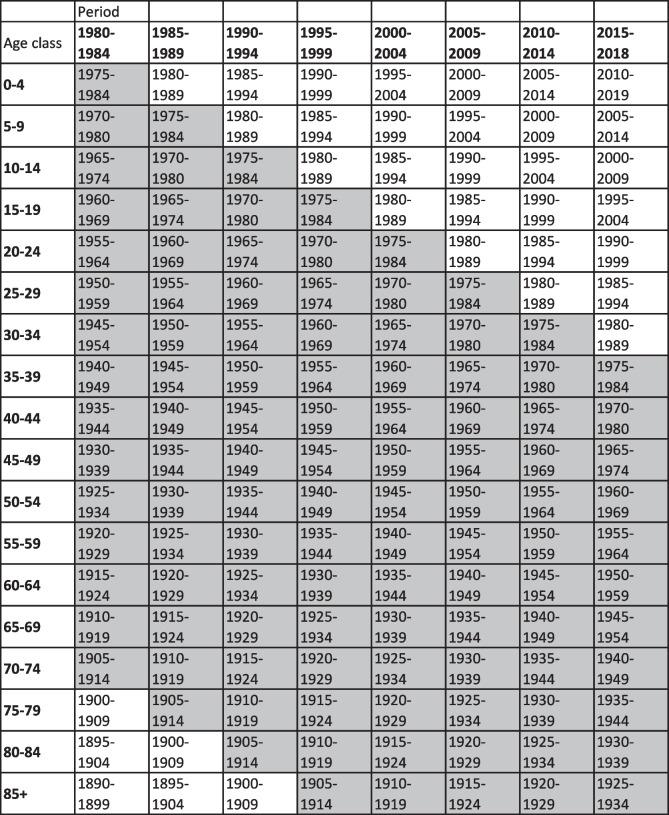
Lexis diagram showing the age groups, the calendar periods and –highlighted in gray– the birth cohorts considered in the analysis

Data were aggregated into 18 five-year age groups (0–4, 5–9, ..., ≥85) and eight five-year periods (with the exception of the last one which was a four-year period: 1980–1984, 1985–1989, 1990–1994, 1995–1999, 2000–2004, 2005–2009, 2010–2014, 2015–2018). Figure 2 shows the Lexis diagram depicting the birth cohorts. The 15 cohorts that were considered in the analysis are highlighted.

The calendar period analysis was performed by calculating the direct standardised rate (2013 European standard population) and the standardised mortality ratio (SMR). The age- and sex-specific rates observed in the population of the three provinces to which the 30 municipalities belong, for the same calendar period, were used as reference.

The birth cohort analysis was performed using the 20–84 years cumulative risk (CR) and the 20–84 years cumulative SMR (SMRcum). Let define O_*ic*_ and *PY*_*ic*_ the number of observed deaths and person years of observation for the i-th age class and c-th birth cohort. We fitted a log-linear Poisson regression model:

$$\log \left({\textrm{O}}_{ic}\right)={\beta}_i+{\beta}_c^{cohort}+\log \left({PY}_{ic}\right)$$

where *β*_*i*_ is the coefficient for the i-th age class (logarithm of the specific i-th age class rate), ${\beta}_c^{cohort}$ is the coefficient for the c-th cohort (logarithm of the ratio between the rate for the c-th cohort and the rate in the reference cohort) and log(*PY*_*ic*_) is the offset, i.e., the weight to be attributed to the observed frequency. For the analysis of the age-cohort model, the central cohort or the one with the largest number of complete data is usually used as reference category. In this study, the cohort of those born in the years 1935–1944 was chosen [60, 61]. The intercept was not included in the model and, consequently, the age-specific coefficients can all be interpreted directly as logarithms of the age-specific rates. The 20–84 years CR for the c-th cohort was obtained as:

$${CR}_c=1-\mathit{\exp}\left(-5{\sum}_{i=20-24}^{I=80-84}\left({\beta}_i+{\beta}_c^{cohort}\right)\right)$$

and its confidence interval (CI) was obtained through the delta method [62] (the results –not shown– are available from the corresponding author upon request). We also calculated the 20–84 years SMRcum for the birth cohorts under study. The regression model was similar to the one described above, but with the logarithm of the number of expected cases being used as offset. For the c-th cohort, the 20–84 years SMRcum was given by:

$$SM{Rcum}_c=\frac{1}{13}{\sum}_{i=20-24}^{I=80-84}\mathit{\exp}\left({\beta}_i+{\beta}_c^{cohort}\right)$$

In this instance, the number of expected cases was obtained for each calendar period on the basis of the age-specific rates in the reference population [63].

Observed deaths (Obs), directly standardised mortality rates (2013 European standard population) per 100,000, 0–59 years and ≥ 60 years cumulative mortality rates, attributable deaths ($Obs\times \frac{SMR-1}{SMR}$) and probability of 0 attributable deaths (q-values calculated considering the total number of causes of deaths and calendar period by sex and area) can be found in Supplementary Table S4.

The analysis of the trend in mortality from testicular cancer was difficult due to a strong increase in survival since 1980. In Italy, an age-period-cohort analysis showed a striking decrease in mortality since 1970 [64]. Due to power considerations, we analysed mortality from testicular cancer only for the period 1980–1999.

All statistical analyses were performed using the version 16 of the Stata statistical package (StataCorp, Texas, USA).

## Results

In the resident population of the *Red area*, during the 34 years from 1985 (assumed date of water contamination) to 2018 (last year of availability of cause-specific mortality), we observed 51,621 deaths vs. 47,731 expected (according to the mortality rates in the three provinces, which include the *Red area*), for an excess of 3890 deaths (90% CI: 3543–4231). In other words, every three days there were 12 deaths vs. 11 expected. The overall SMR was 108 (90% CI: 107–109). Supplementary Table S5A shows the SMR 1985–2018 for all specific causes of death considered. We found evidence for increased mortality from the following conditions: malignant neoplastic diseases (in males); cancer of the liver (in males); pancreas (in males); lung (in males); corpus uteri; kidney and thyroid (in males); diabetes; diseases of the circulatory system, particularly heart diseases and ischemic heart disease; and diseases of the digestive system. In general, all excesses were larger for the population living in the *Red area A*. In a sensitivity analysis, we used as reference the population of the three provinces excluding the 30 municipalities of the *Red area* (Supplementary Table S5B, Supplementary Fig. S8 and S9).

Observed deaths, directly standardised mortality rates (2013 European standard population) per 100,000, 0–59 years and ≥ 60 years cumulative mortality rates, attributable deaths, probability of zero attributable deaths and further details are provided in Supplementary Table S4.

To summarize the time pattern, we compared the SMR for the period 1980–1989 with the overall SMR for the period 1980–2018. This is equivalent to saying that, adopting a conservative approach, we considered the 1980s as a period free of considerable population exposure. We also compared the SMR for the last observed period, 2010–2018, with the overall SMR for the years 1980–2018. In other words, we considered the years 2010–2018 as the period with the highest level of cumulative exposure (Supplementary Table S6). For all causes of death, we found strong evidence for an increased mortality risk vs. the baseline level of the 1980s, an increase that was present even after 2010. This pattern was observed in the *Red area*, but particularly in the *Red area A*, and both in males and females. Mortality from diseases of the circulatory system, heart diseases and ischemic heart disease showed a comparable trend. Diseases of the digestive system and diabetes showed a mortality increase after 2010, although this was not observed in the *Red area A*. Mortality from malignant neoplasms increased after the 1980s in both sexes. The pattern for specific cancer sites was less clear due to a larger statistical uncertainty. We did not find evidence for increasing mortality –by calendar period– from liver, lung, breast, ovarian and thyroid cancers and malignant neoplasms of the lymphohematopoietic system. We found evidence for an increased risk of dying from kidney cancer in both sexes and from testicular cancer.

The excess risk could also vary by birth cohort, being more severe the younger the age at first exposure. If the time trend was linear, however, we could not disentangle the effect of the calendar period from the effect of the birth cohort. Due to statistical considerations regarding the sample size and the number of events, we performed an age-period-cohort analysis of all-cause mortality only. The results showed a greater importance of the birth cohort time axis among males, while for females we found more relevant the calendar period time axis (Table 2). The main results in terms of birth cohort and, then, of calendar period are described below.

**Table 2:** Summaries of fitting different age-period-cohort models to all-cause mortality data from the perfluoroalkyl and polyfluoroalkyl substances (PFAS)-contaminated *Red area*. 1980–2018

	Marginal likelihood ratio (degrees of freedom)	
	Males	Females
Age	147 (125)	131 (125)
Age + drift	144 (124)	126 (124)
Age + drift + cohort	94 (101)	105 (101)
Age + drift + period	117 (118)	109 (118)
Age + drift + cohort + period	87 (95)	95 (95)

The *Red area* includes the 30 municipalities in the provinces of Vicenza, Padua and Verona (northern Italy) connected to the PFAS-contaminated aqueduct

### Birth cohort analysis

#### All-cause mortality

**Fig. 3:**
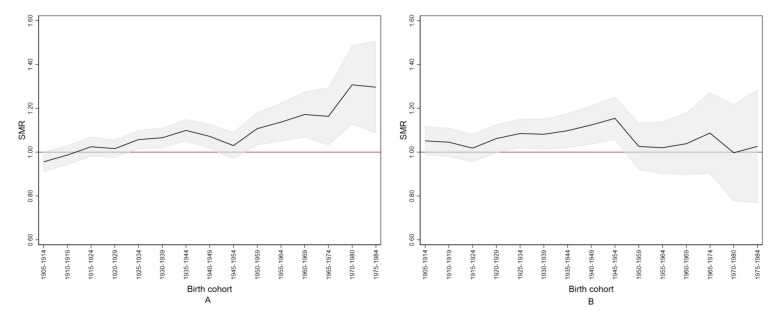
Cumulative standardised mortality ratio (SMR) for all causes by sex and birth cohort (1905–1984) in the perfluoroalkyl and polyfluoroalkyl substances (PFAS)-contaminated *Red area*. Panel (**A**), males; panel (**B**), females. The grey area indicates the 90% confidence band. The red line indicates the reference value. The *Red area* includes the 30 municipalities in the provinces of Vicenza, Padua and Verona (northern Italy) connected to the PFAS-contaminated aqueduct

Figure 3 shows the SMRcum for all-cause mortality. Among females, panel B shows that the SMRcum for all-cause mortality grew from the birth cohort of 1920–1929 and peaked (SMRcum: 1.15; 90% CI: 1.05–1.25) in the birth cohort of 1945–1954, i.e., those females exposed from the age of 35–39 and older. Notably, the SMRcum among females aged less than 35 years in 1984–1989, born after 1960, did not differ from the reference population.

Among males (panel A), the SMRcum rose consistently and almost linearly starting with those born in 1920–1929 and after, with a peak (SMRcum: 1.31; 90% CI: 1.13–1.49) in the birth cohort of 1970–1980. The birth cohort dimension was more important than the calendar period. By the way, comparing the two sexes, the SMRcum in females born until 1945–1954 rose more steeply than in males. Among the latter, a SMRcum of 1.14 (90% CI: 1.05–1.22) was reached by the birth cohort of 1955–1964, i.e., 10 years later than females, who reached a SMRcum of 1.15 (90% CI: 1.05–1.25) with the birth cohort of 1945–1954.

#### Circulatory disease mortality

Similar patterns were also observed for diseases of the circulatory system but with greater statistical uncertainty.

#### Cancer mortality

**Fig. 4:**
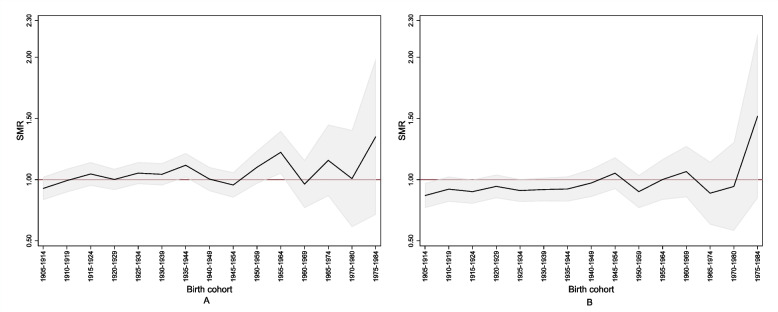
Cumulative standardised mortality ratio (SMR) for malignant neoplasms by sex and birth cohort (1905–1984) in the perfluoroalkyl and polyfluoroalkyl substances (PFAS)-contaminated *Red area*. Panel (**A**), males; panel (**B**), females. The grey area indicates the 90% confidence band. The red line indicates the reference value. The *Red area* includes the 30 municipalities in the provinces of Vicenza, Padua and Verona (northern Italy) connected to the PFAS-contaminated aqueduct

Figure 4 shows that, among females, the SMRcum for mortality from malignant neoplasms grew starting with the birth cohort of 1945–1954 and peaked (SMRcum: 1.52; 90% CI: 0.85–2.19) in the birth cohort of 1975–1984, i.e., the females exposed from the age of 5–9 years. Among males, the SMRcum increased almost linearly starting with those born in 1935–1944 and after, peaking (SMRcum: 1.35; 90% CI: 0.71–1.98) in the birth cohort of 1975–1984, i.e., the males exposed from the age of 5–9 years. Patterns similar to this were also observed for specific cancer sites but with strong statistical uncertainty.

### Calendar period analysis

Supplementary Table S5A shows the observed deaths, the SMR and the 90% CI for each five-year calendar period between 1980 and 2018, by sex and cause of death. Here we point out the most relevant findings.

#### Circulatory disease mortality

**Fig. 5:**
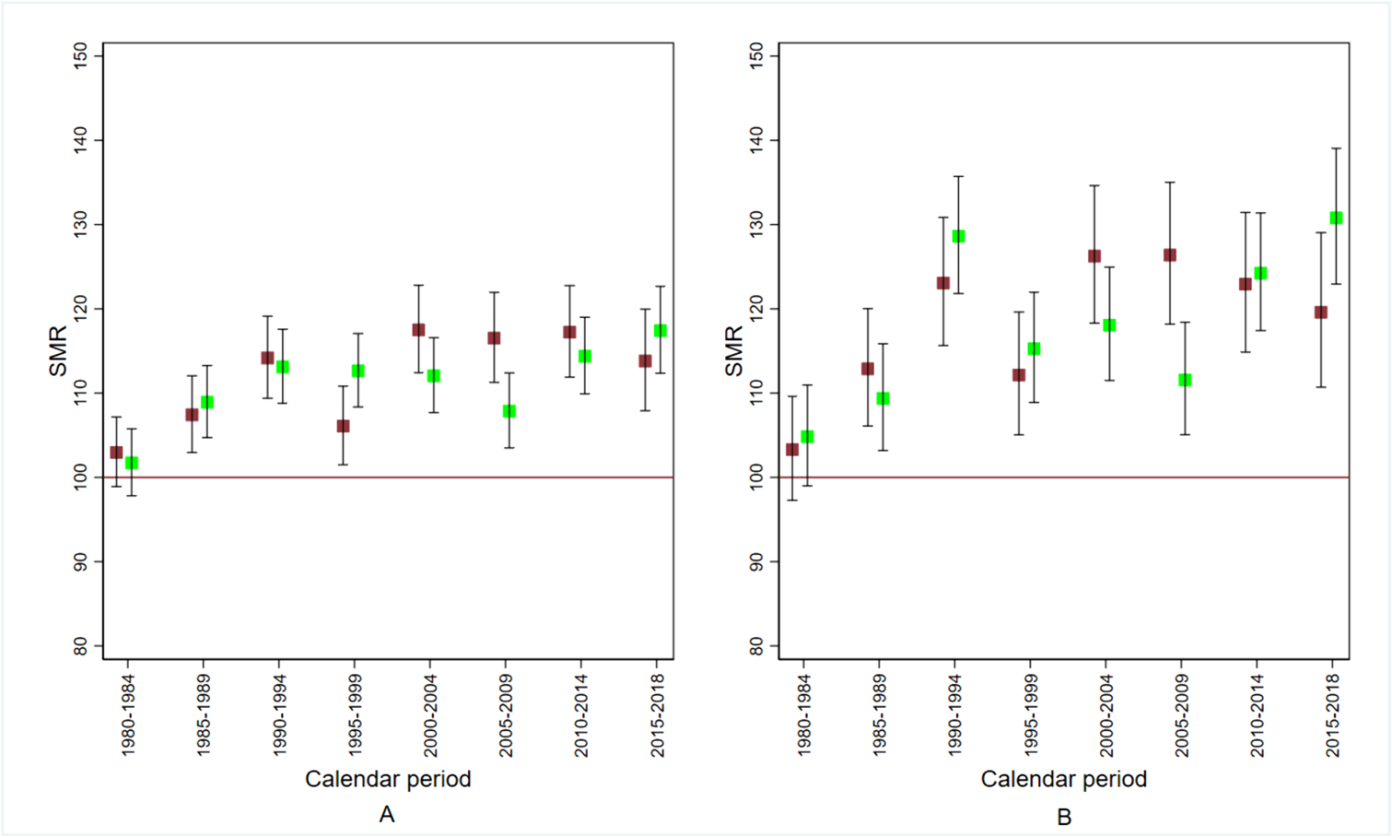
Standardised mortality ratio (SMR) for cardiovascular disease by sex and calendar period in the perfluoroalkyl and polyfluoroalkyl substances (PFAS)-contaminated *Red area*. Red, males; green, females. Panel (**A**), *Red area*; panel (**B**), *Red area A*. The *Red area* includes the 30 municipalities in the provinces of Vicenza, Padua and Verona (northern Italy) connected to the PFAS-contaminated aqueduct. The *Red area A* includes the subset of 13 municipalities with PFAS contamination of the groundwater too. 1980–2018

Figure 5 shows the sex- and cause-specific SMR according to calendar period. The SMR exceeded the null value from the calendar period 1985–1989 onward, in both sexes. There was no evidence for a decrease after 2014. In the calendar period 1980–1984, no excess risk was observed. The SMR in the population of the *Red area A* was higher, peaking in 2015–2018 at 131 (90% CI: 123–139) for females and 120 (90% CI: 111–129) for males.

#### Cancer mortality

With respect to malignant neoplasms (Table 3), we found an increasing trend by calendar period in both sexes. Taking the 1980s (1980–1989) as an internal reference and considering two broad time periods (1990–2009 and 2010–2018), we observed a clear period effect (likelihood ratio, LR: 10.66; degrees of freedom, df: 2; *p*-value = 0.005), without evidence for a difference by sex (LR: 2.18; df: 2; *p*-value = 0.337). Consistently with the long latency period of cancer, an excess risk was observed more than 20 years after the water contamination (see Table 4 for details).

**Table 3:** Observed and expected deaths from malignant neoplasms by sex and calendar period in the perfluoroalkyl and polyfluoroalkyl substances (PFAS)-contaminated *Red area*. 1980–2018

	Males		Females	
Calendar period	Observed	Expected	Observed	Expected
1980–1989	2213	2243	1371	1452
1990–2009	4866	4866	3218	3375
2010–2018	2151	1981	1632	1656

The *Red area* includes the 30 municipalities in the provinces of Vicenza, Padua and Verona (northern Italy) connected to the PFAS-contaminated aqueduct

**Table 4:** Summaries of fitting a Poisson regression model on malignant neoplasms in the perfluoroalkyl and polyfluoroalkyl substances (PFAS)-contaminated *Red area*. 1980–2018

	Mortality rate ratio	90% CI	*p*-value
Females vs. males	0.92	0.89–0.94	< 0.001
1980–1989	Reference		
1990–2009 vs. 1980–1989	1.05	1.01–1.08	0.021
2010–2018 vs. 1980–1989	1.08	1.04–1.12	0.001

*CI* confidence interval

The *Red area* includes the 30 municipalities in the provinces of Vicenza, Padua and Verona (northern Italy) connected to the PFAS-contaminated aqueduct

#### Kidney cancer mortality

**Fig. 6:**
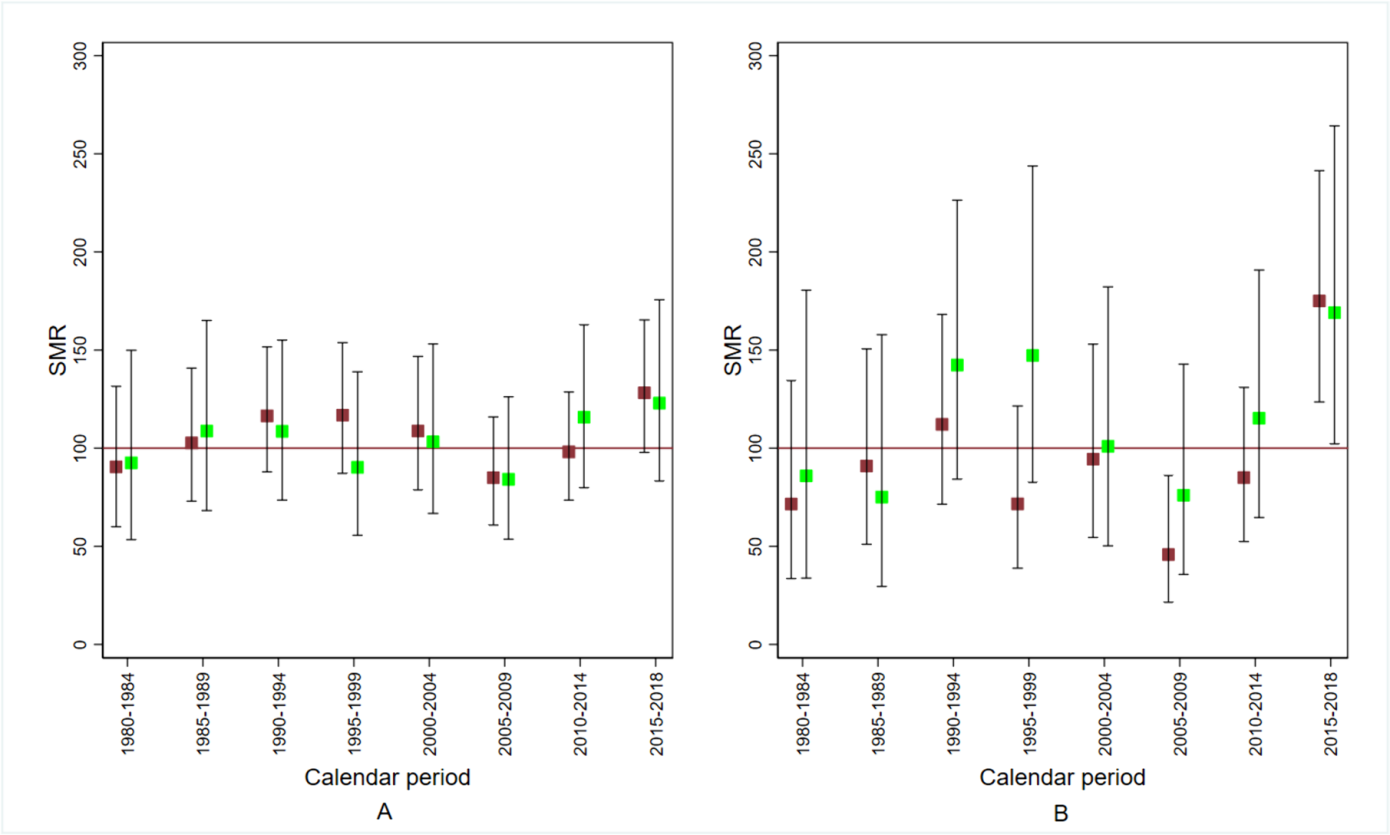
Standardised mortality ratio (SMR) for kidney cancer by sex and calendar period in the perfluoroalkyl and polyfluoroalkyl substances (PFAS)-contaminated *Red area*. Red, males; green, females. Panel (**A**), *Red area*; panel (**B**), *Red area A*. The *Red area* includes the 30 municipalities in the provinces of Vicenza, Padua and Verona (northern Italy) connected to the PFAS-contaminated aqueduct. The *Red area A* includes the subset of 13 municipalities with PFAS contamination of the groundwater too. 1980–2018

Figure 6 shows the sex- and cause-specific SMR according to calendar period. The SMR exceeded the null value from 2015 to 2018 in both sexes. In the period 1980–1984, no excess was found (see also Table 5). This pattern is more evident for the *Red area A* (see Fig. 6, panel B and Supplementary Table S5A).

**Table 5:** Observed and expected deaths from kidney cancer by sex and calendar periods in the perfluoroalkyl and polyfluoroalkyl substances (PFAS)-contaminated *Red area*. 1980–2018

	Males		Females	
Calendar period	Observed	Expected	Observed	Expected
1980–1989	48	49	28	28
1990–2009	138	129	72	74
2010–2018	81	72	46	49

The *Red area* includes the 30 municipalities in the provinces of Vicenza, Padua and Verona (northern Italy) connected to the PFAS-contaminated aqueduct

The age- and sex- SMR for the period 2010–2018 was 1.14 (one-sided mid-*p*-value = 0.066). In the last period observed (2015–2018), the age-sex standardised SMR was 1.26 (90% CI: 1.02–1.54; one-sided mid-*p*-value = 0.034) (see also Supplementary Table S5A). These values were higher in the *Red area A*. The age-sex standardised SMR was 173 (90% CI: 132–221, one-sided mid-*p*-value = 0.0006). The SMR was 175 (90% CI: 124–241) for males and 169 (90% CI: 102–264) for females. In summary, 41 observed deaths vs. 23.7 expected yielded 17 attributable cases in the four-year calendar period 2015–2018, corresponding to four attributable deaths per year in the last period of follow-up.

Noticeably, when restricting the analysis to renal cell kidney cancer (ICD-10 C64 code) for the period 2010–2018 in the *Red area*, we found 38 deaths vs. 26 expected among males, for a SMR of 148 (90% CI: 111–194), and 20 deaths vs. 14 expected among females, for a SMR of 140 (90% CI: 111–204).

#### Testicular cancer mortality

In the time period 1985–1999, we observed six deaths vs. an expected number of 4.3, for a SMR of 140 (90% supported range: 61–275) (mid-*p*-value = 0.204). After 1999, no deaths for testicular cancer were recorded. In the first time period, 1980–1984, we observed one death vs. 2.4 expected.

Restricting the analysis to the population living in the *Red area* A, we found five deaths in the period 1985–1999 vs. 1.95 expected, corresponding to a SMR of 256 (90% CI: 101–539; one-sided mid-*p*-value = 0.032) (Supplementary Table S5A).

## Discussion

### Main findings

In the 30 municipalities connected to the contaminated aqueduct, between 1985 (assumed date of water contamination) and 2018 (last year of availability of cause-specific mortality data), we observed 51,621 deaths vs. 47,731 expected (age- and sex-SMR: 108; 90% CI: 107–109). The overall burden was an excess of 3890 deaths (90% CI: 3543–4231), which is equivalent to saying that, every three days, there were 12 deaths vs. 11 expected. There was evidence for increased mortality from several conditions, namely: malignant neoplastic diseases (in males); cancer of the liver (in males); pancreas (in males); lung (in males); corpus uteri; kidney and thyroid (in males); diabetes; diseases of the circulatory system, particularly heart diseases and ischemic heart disease; and diseases of the digestive system. Comparing the data on the progression of contamination in time and space with the data on the production of specific molecules, it clearly appears that these effects should mainly be attributed to long-chain PFAS, i.e., PFOA and PFOS [40].

The evidence regarding kidney cancer and testicular cancer is consistent with previously reported data. Conversely, this is the first study formally demonstrating an association of PFAS exposure with mortality from cardiovascular disease. Previous research, however, has shown or suggested that PFAS exposure is associated with health conditions –particularly high levels of total cholesterol and low-density cholesterol and posttraumatic stress disorders– that convey an increased risk of cardiovascular disease.

### Interpretation

#### All-cause mortality

With respect to all-cause mortality, both sexes showed an excess risk. As there was strong evidence for an increase in mortality in the most recent birth cohorts, we suggest that this is the effect of PFAS exposure during early human development. Among males, the SMRcum rose almost linearly starting with the birth cohort of 1920–1929 and peaking in the birth cohort of 1970–1980, which reflects the early life exposure and the growing cumulative exposure to a persistent organic pollutant. With respect to females, an interesting finding was that the risk peaked in the birth cohort of 1945–1954, i.e., in females exposed when they were already aged 35 years and older. Females aged less than 35 years in 1984–1989, conversely, did not experience any excess risk. In fact, this protection status was probably accounted for by multiparous females, because nulliparous ones have been demonstrated to have higher PFAS concentrations [65]. It is worthy of note that this has also been observed in the Veneto Region, as can be seen from data in Table 3 and Supplementary Table S1 in the article by Pitter et al. [35]. The protection status of multiparous females is explained by the accumulation of PFAS in the placenta [66], coupled with trans-placental transmission, and by breastfeeding. These mechanisms are the most important determinant of PFAS exposure in early life [67].

#### Circulatory disease mortality

The striking increase in mortality from diseases of the circulatory system, particularly heart diseases and ischemic heart disease, is a valuable finding of this study. Since we used a credible causal inference design, we interpret the results as consequences of PFAS exposure. The effect of PFAS exposure on cardiovascular disease is most likely mediated through the atherosclerotic process. PFOA increases the serum levels of total cholesterol and low-density cholesterol [68]. Evidence has been obtained from cross-sectional studies [23] and longitudinal studies [69]. Notably, several significant associations of PFAS serum levels with multiple markers of cardiovascular disease, including blood pressure [70], levels of triglycerides [70, 71], total cholesterol and low-density cholesterol in children, adolescents [72] and young adults [70], have also been observed in the Veneto Region. PFAS concentrations are primarily associated with blood lipids and apolipoproteins in subspecies of intermediate-, low-, and high-density lipoprotein containing apoC-III, which cause an elevated cardiovascular risk [73]. In more detail, the serum levels of PFOS, PFOA and PFDeA, but not PFHxS, are positively associated with the levels of cholesterol in lipoprotein subfractions, apolipoproteins, and composite fatty acid and phospholipid profiles [74]. The most consistent associations were found for the relationship of PFAS with total cholesterol in intermediate-density lipoprotein, across all low-density lipoprotein subfractions and small high-density lipoprotein.

We raise the hypothesis that a second mechanism leading to an increased risk of cardiovascular disease in PFAS-exposed populations is mediated through the occurrence of posttraumatic stress disorders caused by the severe psychologic trauma. The finding that the risk showed no or minimum decrease after 2014, when contamination mitigation measures and the health surveillance plan were implemented, provides circumstantial evidence for this. It is known that exposure to an environmental contamination resulting from industrial processes or accidents is psychologically stressful for the affected communities [75–77]. Our hypothesis is further supported by a local study which has evaluated the psychosocial impact on people who live in polluted areas and its consequences for the parental role [78]. According to this study, the uncertainty about the health effects of PFAS exposure is a major factor of stress particularly for families with children. This is due to parents’ anxiety regarding the health and the quality of life of their children, combined with the sense of responsibility and the inability to control the destiny of their families [79]. Posttraumatic stress disorders, in turn, are known to be associated with major risk factors for cardiovascular disease, including hypertension and diabetes, and with major cardiovascular disease outcomes such as myocardial infarction and heart failure [80]. In the long term, environmental stressors may lead to allostatic overload, which occurs when stress causes physiological changes and imbalances in stress mediators such as glucocorticoids, excitatory amino acids, and cytokines [81]. Through these processes, chronic stress can further increase the risk of hypertension and ischemic heart disease, which may also make individuals more susceptible to pollutants [82]. Chronic stress itself interacts with exposure to pollutants and amplifies their effects by compromising, for example, the immune system [83].

A third factor could be at play in causing an increased risk of cardiovascular disease in this PFAS-exposed population, i.e., the observed excess risk of diabetes. In our data, we found an increased mortality from diabetes only after 2010 without evidence for a difference in the *Red area A*. This finding may well be independent of PFAS exposure and, consequently, a cautious interpretation is needed. Associations of serum PFAS concentrations with multiple glycemic indicators of type 2 diabetes like glucose and insulin have often been reported, but epidemiological studies have yielded more conflicting results [84]. In the C8 Health Project, the levels of PFAS were negatively associated with diabetes and this inverse relationship was stronger for type 1 diabetes [85]. Melzer et al. found no significant relationship of PFOA and PFOS with self-reported diabetes in the National Health and Nutrition Examination Survey population, and people in the higher quartiles of levels of PFAS had a non-significantly lower risk compared with the first quartile [86]. Conversely, a study from the municipality of Ronneby (southern Sweden), where many residents were exposed to PFAS from drinking water, showed an increased risk of type 2 diabetes [87]. Additionally, it must be considered that diabetes could be underreported as a cause of vascular death. It clearly appears that, based on these inconclusive but interesting observations, the role of diabetes in cardiovascular disease of PFAS-exposed people remains among the priorities of future research, possibly with innovative statistical approaches [84].

The importance of our study lies in the fact that, despite the evidence on atherosclerosis and posttraumatic stress disorders as direct causes of cardiovascular disease, there has been insufficient epidemiologic demonstration so far of an association between PFAS exposure and the final outcome. This might be explained by two confounding effects. First, a mortality study may underestimate the strength of the association due to effective treatment of lipid disorders with statins (marketed at the end of the 1980s), cholesterol absorption inhibitors (marketed since the early 2000s), fibrates and bile acid sequestrants [88, 89]. Second, it has been hypothesised [90] that the anecdotally reported positive association between PFAS exposure and high-density cholesterol [72], coupled with the inverse association between the latter and the risk of cardiovascular disease, may exert a protective role. A notable detail is that, in the study area, an increased level of high-density cholesterol in people exposed to PFAS has actually been demonstrated [70, 72]. Apart from this, the role of unknown confounders in masking the indirect relationship between PFAS exposure and cardiovascular disease has been discussed [91]. Our study, which took advantage of the *natural* experiment in the *Red area*, is the first to indicate a clear role of PFAS exposure on cardiovascular mortality.

It is also necessary to draw attention to the consistency between our results about mortality from circulatory diseases and the ecological study by Mastrantonio et al., conducted in the same geographic area, given the differences in design and identification of the exposed population [92]. They used a report published by the ARPAV in 2015 to define the exposed municipalities and 2013–2014 data from the ARPAV archive to define the unexposed ones (see Fig. 1 which is based on the same data). In particular, they used data on PFAS concentrations in the groundwater, including those under the limit of quantification of 10 ng/L, and extrapolated them to the drinking water supply. The municipalities identified as exposed in this way did not match exactly with those included in the *Red area*.

The coexistence of the increase in mortality from circulatory diseases with a comparable increase in lung cancer mortality among males living in the *Red area* might suggest that both findings depend, at least in part, on a greater exposure to smoking. This hypothesis, however, is contradicted by some interrelated facts: deaths from lung cancer increased only in the *Red area B* (see Supplementary Table S6); the majority of municipalities forming the *Red area B* are situated in the province of Verona (see the ‘Area of water contamination with PFAS’ section); and the trend in lung cancer mortality in the Province of Verona has recently been reported to be less favourable, in both sexes, than that seen in the provinces of Vicenza and Padua [93].

In the *Red area*, incidentally, directly standardised (2013 European standard population) mortality rate for diseases of the circulatory system in 1985–2018, was 35.9 per 10,000 among males and 26.5 among females. These figures exceeded those observed in Italy as a whole in 2019, which were 33.3 and 23.7 per 10,000, respectively (Giada Minelli, personal communication, 2024).

#### Kidney cancer and testicular cancer mortality

The International Agency for Research on Cancer has recently classified PFOA as ‘carcinogenic to humans’ (Group 1) and PFOS as ‘possibly carcinogenic to humans’ (Group 2B) [29]. With respect to single cancer sites, there is ‘limited’ epidemiologic evidence in humans for an association between PFOA exposure and kidney cancer as well as testicular cancer. Our findings add some evidence to the existing literature [29, 94, 95]. As far as kidney cancer is concerned, we found a consistent increase in mortality in the exposed population, which was even more pronounced in the *Red area A*. In a previous ecologic mortality study from the Veneto Region comparing areas with and without PFOA-contaminated drinking water, a moderate excess risk of kidney cancer among females (SMR: 1.32; 95% CI: 1.06–1.65) was found [92]. In a case-control study, nested in a longitudinal cohort using banked blood samples, a positive exposure–response trend for several PFAS including PFOA was observed [96]. An association was also found in an occupational study [33], but these last findings should be considered with caution because of the fact that tetrafluoroethylene is a kidney carcinogen in rodents [97] and that exposure to PFOA and exposure to tetrafluoroethylene were strongly correlated. No animal studies have found an association between PFOA and kidney cancer, but PFOA concentrates in renal tissues. Moreover, long-chain perfluoroalkyl carboxylates induce cytoskeletal abnormalities and activate epithelial–mesenchymal transition in renal cell carcinoma 3D cultures [98]. Overall, this literature evidence suggests that our finding is plausible.

With respect to testicular cancer, mortality is a weak endpoint due to the recent marked improvement in survival from the disease. However, before the year 2000, we found a consistent increase in mortality, particularly in the most exposed population living in the *Red area A*. This is in keeping with the results of two previous studies from the Veneto Region. First, for the period 1997–2014, the analysis of hospital discharge data on orchiectomies for testicular cancer demonstrated a positive association with PFOA exposure at the ecological level [29, 99] and, second, an ecologic mortality study showed an increased risk of disease, although not statistically significant [92]. Elsewhere, an excess risk was reported by a population cohort study with high exposure to PFOA [100] and a case-control study [34]. Both found a clear positive exposure-response. In a nested case-control study, Purdue et al. investigated the association between banked serum PFAS concentrations and the risk of testicular germ cell tumours among U.S. Air Force servicemen [101]. Elevated levels of some PFAS in banked samples were observed for military employment in firefighting. Elevated PFOS concentrations were positively associated with testicular germ cell tumours. Finally, another interesting piece of evidence that goes in the same direction is that PFOA exposure is associated with testicular cancer in rodents [30]. According to a literature review, however, the epidemiologic evidence is supportive but not definitive [90]. Caution was also suggested by a second review [30], because the mechanism by which PFOA causes tumours in rats seems less relevant in humans [102]. According to a meta-analysis, instead, the link with testicular cancer is probably a causal one [103].

In part, the uncertainty regarding testicular cancer reflects methodological constraints. Case ascertainment in the evaluation of the risk of disease is almost certainly biased downward if the endpoint is mortality, because advancements in therapy have greatly improved long-term survival in most patients [104]. While in North-America mortality has been very low and virtually stable since 1980 [105], the Italian rate has decreased rapidly after 1970 [64]. For this reason, we restricted the analysis to the last two decades of the past century. We assume, however, that even in the years before 2000 there was a proportion of patients cured of testicular cancer. This introduces a limitation into the study.

### Comparison with previous research from the area of contamination

Several previous studies have evaluated the health surveillance data collected in the *Red area*. The degree of consistency between their results and our own is considerable. Statistically significant associations of serum levels of PFAS with blood pressure and levels of triglycerides have been observed [71]. In children and adolescents, PFOA, PFOS, PFHxS and PFNA were associated with total cholesterol, low-density lipoprotein cholesterol and, to a lesser extent, high-density lipoprotein cholesterol [72]. In young adults, PFOA, PFOS, and PFHxS were associated with total cholesterol, low-density lipoprotein cholesterol and high-density lipoprotein cholesterol, with PFOA and PFHxS being also associated with triglycerides [70]. An association between the serum levels of PFOA, PFOS, PFHxS and PFNA and multiple markers of cardiovascular risk was also found in a subgroup of 232 males formerly employed in the factory of Trissino and attending the health surveillance programme [106]. Another study suggested an association between PFAS concentrations and lipid profiles in pregnant women, with the latter varying by trimester of pregnancy [107]. This relationship is consistent with a great deal of literature from other populations that has reported on the influence of PFAS exposure on lipid metabolism during pregnancy [108, 109].

The current medical literature on the PFAS contamination occurring in the Veneto region also includes an ecological mortality study showing higher rates of multiple causes of death in the contaminated municipalities [92] and an occupational cohort study, which found an association with the cumulative internal dose of PFOA and an increased risk of liver cancer, liver cirrhosis, diabetes, and haematologic malignancies in 462 male workers employed in the factory of Trissino [40]. Finally, a laboratory study has suggested an additional causal role of PFAS in the atherosclerosis process, mediated through an impaired downstream signaling of platelets’ activation and aggregation [110].

### Other environmental contaminants

To complete information on the study area, mention should be made of the fact that the groundwater system in which the PFAS contamination episode occurred has been infiltrated by other pollutants. In particular, episodes of groundwater contamination by organo-halogenated solvents have been documented in 1977, 1987, 1992, 2003 and 2004 [111].

### Methodological issues

Some methodological issues of this study need to be considered. First, the study was based on aggregate data at the municipality level and the interpretation of the results depends on the assumption of homogeneity of exposure within municipalities, which is almost certain for the population of the *Red area A*. The choice of using the population of the provinces of Vicenza, Padua and Verona as reference could have biased conservatively the results. However, a sensitivity analysis conducted excluding the exposed population from the reference gave similar results (Supplementary Table S5B, Supplementary Fig. S8 and S9). In addition, all time period comparisons were done taking as reference the time periods preceding the relevant exposure. This internal analysis reassures in terms of comparability of exposed and unexposed, and the use of SMR guarantees that the confounding by the secular trend in the time evolution of mortality was controlled for.

Second, we could not adjust the association of PFAS exposure with the risk of cardiovascular disease for those conditions acting as mediators of the effect of PFAS, for example low-density cholesterol and hypertension. It must be carefully considered, however, that the adjustment would eclipse the indirect effect of PFAS, which depends on mediating factors.

Third, our period of observation began approximately 15 years after the onset of exposure because of limitations in availability of mortality data. As a consequence, we excluded from the exposed population those municipalities with groundwater contamination since 1966 (the municipality of Trissino and the so called *Orange area* [47]). The inclusion of these municipalities in the reference population may have led to an underestimation of the risk.

And fourth, comparisons with other studies from the same area should be made with some caution because of the possibility that the municipalities considered to represent the ‘contaminated area’ and the uncontaminated (or reference) area have been selected using data from different reports of regional authorities or have been defined according to criteria partly different from ours. The selection of the uncontaminated area is particularly prone to arbitrary assumptions and between-studies variation, with a risk of misclassification of exposure. We took the reference municipalities within the boundaries of the three provinces involved. They probably had some, albeit low, degree of contamination with PFAS but they were much more similar to the contaminated municipalities for many potential confounders.

### Future perspectives

Global concerns about the public health impact of PFAS are growing and the scientific, political and social attitudes towards the PFAS problem are evolving. The revision of the IARC classification of PFOA to ‘carcinogenic to humans’ (Group 1) and the confirmation of PFOS as ‘possibly carcinogenic to humans’ (Group 2B) [29] will most likely boost this process. In the first place, the transition to the replacement of PFAS and the creation of comprehensive environmental monitoring programmes will become important topics in many agendas. The combination of regulative restrictions and pan-European biomonitoring programmes is considered the key strategy for PFAS control across the European Union [28]. Increasing attention is also being paid to the creation of groundwater parks, i.e., areas free of agricultural activities and nutrient-rich sewage sludge.

Although effective strategies for removing PFAS do exist [4], research efforts on technologies and water treatment systems should continue [112]. PFAS treatment and remediation costs, too, have become a dominant topic of environmental policies, and the pressure to find effective and economical solutions is increasing [113, 114].

The exploration of options for PFAS control plans is paralleled by research efforts and advancements on the mechanisms of carcinogenicity. An emerging view is that there are distinct but interdependent general pathways of PFAS action involving, in particular, metabolism, endocrine disruption and epigenetic perturbation [115]. These three pathways seem mutually reinforcing and may combine to establish a pro-tumorigenic environment. Also, these pathways are predicted to be dependent on the dose and the window of exposure during life. This might contribute to explain the difficulties in obtaining epidemiologic and experimental evidence linking PFAS and cancer. The relationship between prenatal and postnatal exposure to PFAS, inflammation status and cardiometabolic factors is another promising area of research [116].

As a final remark, our findings support the view that health surveillance programmes should give more consideration to the psychological impact of environmental pollution, which is poorly recognised by the health authorities responsible for managing disasters [78].

## Conclusions

For the first time, the association of PFAS with mortality from cardiovascular disease was formally demonstrated in the world’s largest exposed population. The evidence regarding kidney cancer and testicular cancer is consistent with previously reported data. Given the present results and the recent IARC revision, it is urged to have an immediate ban of PFAS production and to start implementing additional remediation activities in contaminated areas.

### Supplementary Information

**Supplementary Material 1.**

**Supplementary Material 2.**

## Data Availability

Research data are available from Giada Minelli (contact email: giada.minelli@iss.it) upon reasonable request.

## Abbreviations

ARPAV: Environmental Prevention and Protection Agency of the Veneto Region
CI: Confidence interval
CR: Cumulative risk
df: degrees of freedom
IARC: International Agency for Research on Cancer
IRSA–CNR: Institute of Water Research of the Italian National Research Council (Italian: Istituto di Ricerca Sulle Acque–Consiglio Nazionale delle Ricerche)
ICD: International Classification of Diseases
ISS: Italian National Institute of Health (Italian: Istituto Superiore di Sanità)
ISTAT: Italian National Institute of Statistics (Italian: Istituto Nazionale di Statistica)
LR: Likelihood ratio
Obs: Observed deaths
PD: Padua
PFAS: Perfluoroalkyl and polyfluoroalkyl substances
PFBA: Perfluorobutanoic acid
PFBS: Perfluorobutanesulfonic acid
PFDeA: Perfluorodecanoic acid
PFDoA: Perfluorododecanoic acid
PFHpA: Perfluoroheptanoic acid
PFHxA: Perfluorohexanoic acid
PFHxS: Perfluorohexanesulfonic acid
PFNA: Perfluorononanoic acid
PFOA: Perfluorooctanoic acid
PFOS: Perfluorooctanesulfonic acid
PFPeA: Perfluoropentanoic acid
PFUnA: Perfluoroundecanoic acid
SMR: Standardised mortality ratio
SMRcum: Cumulative standardised mortality ratio
VI: Vicenza
VR: Verona
