# Supplementary material for: All-cause, cardiovascular disease and cancer mortality in the population of a large Italian area contaminated by perfluoroalkyl and polyfluoroalkyl substances (1980–2018)
Source: Environ Health. 2024 Apr 16;23:42. doi: 10.1186/s12940-024-01074-2 (PMC11022451; doi:10.1186/s12940-024-01074-2)
Supplement: Supplementary file 2 — Supplementary Material 2. [file 12940_2024_1074_MOESM2_ESM.pdf]

All-cause, cardiovascular disease and cancer mortality in the population of a large Italian area contaminated by perfluoroalkyl and polyfluoroalkyl substances (1980-2018)

Annibale Biggeri[1], Giorgia Stoppa[1], Laura Facciolo[2], Giuliano Fin[2], Silvia Mancini[3], Valerio Manno[4], Giada Minelli[4], Federica Zamagni[3,*], Michela Zamboni[2], Dolores Catelan[1], Lauro Bucchi[3]

[1] Unit of Biostatistics, Epidemiology and Public Health, Department of Cardiac, Thoracic, Vascular Sciences and Public Health, University of Padua, Padua, Italy

[2] *Comitato mamme NO-PFAS,* Vicenza, Padua, Verona, Italy

[3] Emilia-Romagna Cancer Registry, Romagna Cancer Institute, IRCCS Istituto Romagnolo per lo Studio dei Tumori (IRST) Dino Amadori, Meldola, Forlì, Italy

[4] Statistical Service, Istituto Superiore di Sanità, Rome, Italy

Correspondence
Federica Zamagni, Emilia-Romagna Cancer Registry, Romagna Cancer Institute, IRCCS Istituto Romagnolo per lo Studio dei Tumori (IRST) Dino Amadori, 47014 Meldola, Forlì, Italy.
Email: federica.zamagni@irst.emr.it

Supplementary materials
Supplementary Table S1
Supplementary Table S2
Supplementary Table S3
Supplementary Table S4
Supplementary Table S5A
Supplementary Table S5B
Supplementary Table S6
Supplementary Fig. S1
Supplementary Fig. S2
Supplementary Fig. S3
Supplementary Fig. S4
Supplementary Fig. S5
Supplementary Fig. S6
Supplementary Fig. S7
Supplementary Fig. S8
Supplementary Fig. S9

Supplementary Table S1. Distribution of perfluorooctanoic acid (PFOA) serum concentrations (ng/mL) by attained age at the first invitation to participate in the health surveillance plan and year of invitation. The numbers in each cell are (from the top to the bottom) the number of subjects, the 5th percentile, the mean (in bold), the median and the 95th percentile. Empty cells indicate a number of subjects <50. Data are from the perfluoroalkyl and polyfluoroalkyl substances (PFAS)-contaminated *Red area*.

**Age group (years)**	**Year of invitation**		
	**2017-2018**	**2019-2020**	**2021-2023**
**58-72**		3480<br>9.8<br>**111.3**<br>78.5<br>309.8	7263<br>3.2<br>**81.9**<br>50.4<br>265.3
**45-61**	1472<br>9.1<br>**91.8**<br>64.7<br>264.7	8443<br>3.6<br>**71.2**<br>41.1<br>240.7	1771<br>3.1<br>**58.9**<br>34.3<br>187.0
**36-51**	7042<br>3.6<br>**62.0**<br>33.7<br>217.7	4841<br>2.3<br>**42.8**<br>22.1<br>149.0	78<br>1.2<br>**26.6**<br>12.9<br>112.2
**26-41**	7783<br>3.3<br>**60.5**<br>34.8<br>203.3	847<br>1.5<br>**31.4**<br>15.1<br>118.7	100<br>0.4<br>**20.8**<br>8.5<br>93.8
**15-31**	8721<br>7.2<br>**65.2**<br>50.6<br>170.3	899<br>2.5<br>**28.6**<br>20.1<br>81.4	263<br>0.8<br>**15.9**<br>9.8<br>48.5
**15-17**		1848<br>4.2<br>**28.7**<br>24.2<br>67.0	
**14-18**		96<br>2.2<br>**21.9**<br>19.0<br>52.0	1167<br>1.6<br>**12.9**<br>9.9<br>33.0
**14-16**			660<br>1.7<br>**9.8**<br>7.9<br>22.4
**8-12**	182<br>7.7<br>**30.7**<br>25.8<br>69.1	1713<br>4.6<br>**26.8**<br>21.7<br>64.4	
**8-13**		873<br>3.9<br>**23.6**<br>17.9<br>64.2	552<br>1.2<br>**7.8**<br>5.6<br>23.6

Supplementary Table S1. Continued

Age group (years)	Year of invitation		
	**2017-2018**	**2019-2020**	**2021-2023**
**8-11**			746
			1.1
			**7.8**
			5.0
			22.5
**7-9**			257
			1.2
			**8.2**
			4.9
			24.4

Note: The *Red area* includes the 30 municipalities in the provinces of Vicenza, Padua and Verona (northern Italy) connected to the PFAS-contaminated aqueduct. 2017-2023. Source: Regione del Veneto. Piano di sorveglianza sanitaria sulla popolazione esposta a PFAS. Rapporto n. 17. Maggio 2023 (https://elezioni.regione.veneto.it/documents/10793/12935055/Bollettino+PFAS+n.+17+-+maggio+2023.pdf/95cd8c4c-8790-4725-b5b0-f3cd089b51cc. Accessed 14 Feb 2024)

Supplementary Table S2. Distribution of perfluorooctanesulfonic acid (PFOS) serum concentrations (ng/mL) by attained age at first invitation to participate in the health surveillance plan and year of invitation. The numbers in each cell are (from the top to the bottom) the number of subjects, the 5th percentile, the mean (in bold), the median and the 95th percentile. Empty cells indicate a number of subjects <50.
Data are from the perfluoroalkyl and polyfluoroalkyl substances (PFAS)-contaminated *Red area*.

**Age group (years)**	**Year of invitation**		
	**2017-2018**	**2019-2020**	**2021-2023**
**58-72**		3480	7263
		2.0	1.6
		**6.2**	**6.6**
		5.3	5.3
		13.1	15.3
**45-61**	1472	8443	1771
	1.6	1.4	1.4
	**5.5**	**5.8**	**5.2**
	4.7	4.6	4.1
	12.0	13.8	12.1
**36-51**	7042	4841	78
	1.2	1.3	0.7
	**4.8**	**4.8**	**4.3**
	3.8	3.7	3.5
	11.0	11.4	10.2
**26-41**	7783	847	100
	1.3	1.2	0.6
	**4.7**	**3.8**	**3.0**
	3.8	3.0	2.3
	10.5	9.3	7.6
**15-31**	8721	899	263
	1.5	1.1	0.6
	**4.7**	**3.3**	**2.9**
	3.8	2.7	2.5
	10.3	7.2	6.1
**15-17**		1848	
		1.1	
		**2.9**	
		2.5	
		6.2	
**14-18**		96	1167
		1.0	0.7
		**2.1**	**2.1**
		1.8	1.8
		4.3	4.5
**14-16**			660
			0.7
			**2.1**
			1.7
			4.4
**8-12**	182	1713	
	1.1	0.9	
	**3.3**	**2.4**	
	2.4	2.0	
	5.4	4.9	
**8-13**		873	552
		1.1	0.4
		**2.8**	**1.6**
		2.4	1.3
		5.6	3.7

Supplementary Table S2. Continued

Age group (years)	Year of invitation		
	**2017-2018**	**2019-2020**	**2021-2023**
**8-11**			746
			0.5
			**1.7**
			1.4
			4.0
**7-9**			257
			0.5
			**1.6**
			1.2
			4.2

Note: The *Red area* includes the 30 municipalities in the provinces of Vicenza, Padua and Verona (northern Italy) connected to the PFAS-contaminated aqueduct.2017-2023. Source: Regione del Veneto. Piano di sorveglianza sanitaria sulla popolazione esposta a PFAS. Rapporto n. 17. Maggio 2023 (https://elezioni.regione.veneto.it/documents/10793/12935055/Bollettino+PFAS+n.+17+-+maggio+2023.pdf/95cd8c4c-8790-4725-b5b0-f3cd089b51cc. Accessed 14 Feb 2024)

Supplementary Table S3. Summary of the ICD-9 and ICD-10 (2019 version) codes used in the study with details of the causes of death considered

ICD-9	ICD-10	Cause of death	Details
001-999	A00-Z99.9	All causes	
140-208	C00-C97	Malignant neoplasms	All malignant neoplasms, including the categories analysed in detail in this analysis
155	C22	Malignant neoplasm of liver and intrahepatic bile ducts	Malignant neoplasm of liver and intrahepatic bile ducts, excluding gallbladder and extrahepatic bile ducts
155.0	C22.0, C22.2	Malignant neoplasm of liver	Malignant neoplasm of liver including liver cell carcinoma and hepatoblastoma
157	C25	Malignant neoplasm of pancreas	Malignant neoplasm of pancreas
162	C33-C34	Malignant neoplasm of trachea, bronchus and lung	Malignant neoplasm of trachea, bronchus and lung excluding pleura
174	C50	Malignant neoplasm of breast	Malignant neoplasms of female breast
182	C54	Malignant neoplasm of corpus uteri	Malignant neoplasm of corpus uteri
183	C56-C57	Malignant neoplasms of ovary and other and unspecified female genital organs	Malignant neoplasms of ovary and other and unspecified female genital organs. They include Fallopian tube, broad ligament, round ligament and parametrium
186	C62	Malignant neoplasm of testis	Malignant neoplasm of testis. They include malignant tumour of undescended, descended and unspecified testis
189	C64-C66, C68	Malignant neoplasms of kidney and other and unspecified urinary organs	Malignant neoplasm of kidney, renal pelvis, ureter, urethra, paraurethral glands and contiguous or overlapping sites, excluding bladder
193	C73	Malignant neoplasm of thyroid gland	Malignant neoplasms of thyroid gland
200-208	C81-C96	Malignant neoplasms of lymphoid, haematopoietic and related tissue	All solid and non-solid malignant tumours of the lymphatic and hematopoietic tissue
250	E10-E14	Diabetes mellitus	Diabetes mellitus including acute problems (e.g. hypoglycaemic coma, ketoacidosis) and complications (renal or ophthalmic complications, neurological and peripheral circulatory complications)
390-459	I00-I99	Diseases of the circulatory system	Including the categories of heart disease described below, cerebrovascular disease, diseases of the arteries (atherosclerosis, aneurysms, embolisms and vasculitis), veins (thrombosis and embolisms) and lymphatic vessels
390-429	I00-I09, I11, I13, I20-I51	Heart diseases	Including rheumatic, hypertensive, and ischaemic heart diseases described below, pulmonary circulation diseases, pericardial, endocardial and myocardial disease, conduction disorders and heart failure
410-414	I20-I25	Ischaemic heart diseases	Ischaemic heart diseases
520-579	K00-K93	Diseases of the digestive system	Diseases of the digestive system

Abbreviation: *ICD-9* International classification of diseases, 9th revision; *ICD-10* International classification of diseases, 10th revision

Supplementary Table S4. See file Supplementary Table S4.xlsx

Supplementary Table S5A. Observed number of deaths (Obs), standardised mortality ratios (SMR) and 90% confidence interval (CI) by cause of death, area, sex and calendar period. The *Red area* includes the 30 municipalities of the Veneto Region connected to the perfluoroalkyl and polyfluoroalkyl substances (PFAS)-contaminated aqueduct. The *Red area A* includes the subset of 13 municipalities with PFAS contamination of the groundwater too. The reference population is the population of the three provinces of Vicenza, Padua and Verona (northern Italy). 1980-2018

			**1980-1984**		**1985-1989**		**1990-1994**		**1995-1999**		**2000-2004**		**2005-2009**		**2010-2014**		**2015-2018**		**1980-2018**		**1985-2018**	
**Cause of death (ICD9 code)**	**Area**	**Sex**	**Obs**	**SMR (90% CI)**	**Obs**	**SMR (CI 90%)**	**Obs**	**SMR (90% CI)**	**Obs**	**SMR (90% CI)**	**Obs**	**SMR (90% CI)**	**Obs**	**SMR (90% CI)**	**Obs**	**SMR (90% CI)**	**Obs**	**SMR (90% CI)**	**Obs**	**SMR (90% CI)**	**Obs**	**SMR (90% CI)**
All causes (001-999)	*Red area*	M	4037	104 (101-106)	3957	108 (105-111)	3833	108 (105-111)	3729	106 (103-109)	3663	111 (108-114)	3539	107 (104-110)	3763	113 (110-116)	3108	113 (110-116)	29629	108 (107-109)	25592	109 (108-110)
		F	3489	104 (101-107)	3546	106 (103-109)	3580	106 (103-109)	3652	106 (103-109)	3627	104 (102-107)	3719	105 (102-108)	4211	110 (107-113)	3694	113 (110-116)	29518	107 (106-108)	26029	107 (106-108)
	Red area A	M	1839	105 (101-109)	1808	110 (105-114)	1781	113 (108-117)	1717	111 (107-116)	1641	112 (107-117)	1603	109 (105-114)	1732	114 (109-118)	1459	116 (111-121)	13580	111 (109-112)	11741	112 (110-114)
		F	1659	108 (104-113)	1615	105 (101-110)	1766	115 (111-120)	1717	109 (105-113)	1733	108 (104-112)	1746	106 (102-111)	2009	114 (110-118)	1773	118 (113-123)	14018	110 (109-112)	12359	110 (109-112)
Malignant neoplasms (140-208)	*Red area*	M	1075	100 (96-106)	1238	106 (101-111)	1265	106 (101-111)	1223	107 (102-112)	1220	109 (104-114)	1158	101 (96-106)	1221	109 (104-114)	930	108 (103-114)	9330	106 (104-108)	8255	106 (105-108)
		F	642	95 (89-102)	729	94 (89-100)	754	93 (87-99)	784	95 (89-101)	795	94 (89-100)	885	99 (94-105)	907	99 (94-105)	725	98 (92-104)	6221	96 (94-98)	5579	96 (94-98)
	Red area A	M	444	93 (86-101)	517	99 (92-107)	557	106 (98-113)	540	107 (99-115)	527	106 (98-114)	475	93 (86-100)	538	106 (98-114)	426	108 (100-117)	4024	102 (99-105)	3580	103 (101-106)
		F	287	95 (86-104)	301	86 (78-95)	353	96 (88-105)	370	99 (91-108)	371	96 (88-105)	411	101 (93-109)	411	98 (90-106)	330	96 (88-106)	2834	96 (93-99)	2547	96 (93-99)
Malignant neoplasm of liver and intrahepatic bile ducts (155)	*Red area*	M	58	123 (98-153)	66	105 (84-128)	83	105 (87-127)	94	122 (102-144)	86	110 (91-132)	80	100 (82-120)	93	116 (97-137)	64	107 (86-131)	624	110 (103-118)	566	109 (102-117)
		F	22	79 (54-113)	37	120 (89-158)	31	91 (66-122)	43	113 (86-146)	35	97 (71-128)	42	117 (89-152)	37	98 (73-129)	27	99 (70-137)	274	102 (92-113)	252	105 (94-117)
	Red area A	M	25	119 (83-166)	29	104 (74-141)	35	101 (75-134)	38	112 (84-147)	46	133 (102-170)	42	117 (89-151)	43	118 (90-152)	26	94 (66-131)	284	113 (102-124)	259	112 (101-124)
		F	6	48 (21-94)	16	114 (71-173)	15	97 (60-150)	20	117 (78-171)	18	109 (71-162)	25	153 (106-214)	12	70 (40-113)	6	48 (21-94)	118	97 (83-113)	112	103 (87-120)
Malignant neoplasm of liver (155.0)	*Red area*	M	37	122 (92-161)	34	96 (71-128)	53	98 (77-123)	45	97 (75-1225)	38	94 (70-124)	34	100 (74-133)	51	111 (87-140)	29	117 (84-159)	321	103 (94-131)	284	101 (91-111)
		F	13	82 (48-130)	13	107 (64-171)	17	96 (61-144)	21	125 (84-180)	12	81 (47-132)	15	123 (76-189)	13	95 (56-152)	7	94 (44-176)	111	100 (85-118)	98	104 (87-122)
	Red area A	M	16	120 (75-182)	12	77 (44-125)	26	109 (76-151)	23	113 (77-160)	19	105 (69-154)	22	144 (98-206)	24	114 (79-160)	12	105 (60-170)	154	111 (97-126)	138	110 (95-127)
		F	4	73 (19-127)	4	73 (25-167)	6	76 (33-149)	13	173 (102-275)	5	74 (29-157)	8	144 (72-260)	7	112 (53-210)	<3	28 (1-136)	48	96 (74-122)	44	103 (79-132)
Malignant neoplasm of pancreas (157)	*Red area*	M	50	125 (97-158)	52	108 (84-136)	66	117 (94-144)	57	105 (83-131)	64	104 (84-128)	66	103 (83-126)	96	128 (107-151)	63	102 (82-125)	514	112 (104-120)	464	110 (102-119)
		F	27	81 (57-112)	44	98 (75-126)	39	73 (55-96)	44	77 (59-99)	64	95 (76-117)	67	90 (73-110)	84	105 (87-126)	63	93 (74-114)	432	90 (83-98)	405	91 (84-99)
	Red area A	M	21	118 (79-170)	23	108 (74-153)	28	113 (80-155)	25	105 (73-147)	30	109 (72-137)	29	101 (72-138)	45	132 (101-169)	26	91 (64-127)	227	110 (98-123)	206	109 (97-123)
		F	13	87 (51-138)	13	64 (38-101)	17	71 (45-107)	14	54 (71-133)	30	98 (71-133)	31	91 (71-133)	46	125 (97-160)	28	89 (63-122)	192	88 (78-99)	179	88 (78-99)
Malignant neoplasm of trachea- bronchus and lung (162)	*Red area*	M	339	98 (90-108)	402	108 (100-118)	461	123 (114-133)	366	105 (96-114)	359	111 (101-121)	341	110 (100-120)	304	110 (100-121)	235	114 (102-127)	2807	110 (106-113)	2468	111 (108-115)
		F	49	87 (68-111)	49	76 (59-96)	60	82 (65-101)	68	79 (64-96)	78	80 (66-97)	105	101 (85-118)	105	89 (76-105)	93	98 (82-116)	607	87 (82-93)	558	87 (81-94)
	Red area A	M	137	90 (78-104)	167	102 (89-116)	181	110 (97-124)	165	107 (94-122)	149	104 (90-119)	125	90 (77-105)	131	104 (90-121)	96	101 (85-120)	1151	101 (96-106)	1014	103 (98-108)
		F	25	99 (69-138)	23	79 (54-112)	24	73 (50-102)	36	93 (69-123)	30	68 (49-92)	50	105 (82-133)	50	93 (72-117)	43	97 (74-126)	281	89 (80-98)	256	88 (79-98)
Malignant neoplasm of breast (174)	*Red area*	F	141	112 (100-125)	143	99 (89-111)	146	96 (86-108)	155	98 (88-109)	110	76 (67-86)	159	105 (94-116)	153	102 (91-113)	124	98 (86-110)	1131	98 (93-102)	990	96 (91-102)
	Red area A	F	64	113 (96-134)	62	96 (81-113)	69	101 (86-119)	80	112 (96-130)	44	67 (54-82)	74	106 (91-124)	70	101 (86-118)	69	117 (99-137)	532	101 (94-109)	468	99 (92-107)
Malignant neoplasm of corpus uteri (182)	*Red area*	F	5	79 (31-166)	6	108 (47-213)	12	135 (78-218)	6	78 (34-153)	11	119 (67-197)	7	76 (36-142)	9	91 (47-158)	10	116 (63-197)	66	101 (81-124)	61	103 (82-128)
	Red area A	F	4	140 (48-322)	3	120 (32-309)	6	150 (65-296)	<3	57 (10-181)	5	119 (47-250)	4	95 (32-216)	3	65 (18-169)	<3	50 (9-157)	29	97 (70-134)	25	93 (64-129)
Malignant neoplasm of ovary (183)	*Red area*	F	30	103 (74-140)	40	101 (76-131)	32	78 (57-105)	42	114 (87-147)	41	106 (81-138)	38	97 (73-127)	37	92 (69-121)	41	119 (90-154)	301	101 (91-111)	271	100 (91-111)
	Red area A	F	11	85 (48-140)	19	107 (70-157)	11	60 (34-99)	17	102 (65-154)	20	114 (76-166)	13	72 (43-115)	18	97 (63-144)	14	87 (53-136)	123	91 (78-105)	112	91 (77-107)
Malignant neoplasm of testis (186)	*Red area*	M	<3	42 (2-198)	6	140 (61-275)							<3	0 (0-72)					7	71 (33-132)	6	80 (35-157)
	Red area A	M	<3	93 (4-539)	5	256 (101-539)							<3	0 (0-151)					6	131 (57-260)	5	144 (56-302)
Malignant neoplasm of kidney and other and unspecified urinary organs (189)	*Red area*	M	20	91 (60-132)	28	103 (73-141)	40	116 (88-152)	37	117 (87-154)	31	109 (79-147)	29	85 (61-116)	38	98 (73-129)	43	128 (98-165)	266	106 (96-118)	246	108 (97-120)
		F	12	93 (53-150)	16	109 (68-165)	22	109 (74-155)	15	90 (56-139)	18	103 (67-153)	17	84 (54-126)	24	116 (80-163)	22	123 (83-176)	146	104 (90-119)	134	105 (90-121)
	Red area A	M	7	72 (34-134)	11	91 (51-151)	17	112 (71-168)	10	72 (39-121)	12	94 (54-153)	7	46 (21-86)	15	85 (52-131)	27	175 (124-241)	106	95 (80-111)	99	97 (87-108)
		F	5	86 (34-180)	5	75 (30-158)	13	142 (84-226)	11	147 (83-244)	8	101 (50-182)	7	76 (36-143)	11	115 (65-191)	14	169 (102-264)	74	116 (94-140)	69	119 (96-145)
Malignant neoplasm of the thyroid gland (193)	*Red area*	M	3	107 (29-276)	<3	33 (1-154)	<3	84 (15-265)	<3	42 (2-197)	4	159 (54-363)	<3	86 (15-270)	<3	31 (1-146)	3	146 (45-428)	17	83 (53-124)	14	78 (48-123)
		F	5	92 (36-193)	7	120 (56-226)	4	61 (21-139)	4	76 (26-174)	8	147 (73-266)	<3	48 (8-152)	<3	45 (8-141)	3	80 (22-208)	35	86 (63-114)	30	85 (91-111)
	Red area A	M	<3	81 (3-384)	<3	74 (3-348)	<3	191 (33-601)	<3	94 (4-447)	4	357 (122-817)	<3	96 (4-452)	<3	68 (3-323)	<3	120 (5-569)	12	131 (76-212)	11	139 (78-230)
		F	<3	81 (14-255)	3	114 (31-294)	3	101 (27-262)	3	126 (34-326)	3	121 (33-313)	<3	53 (2-250)	<3	0 (0-113)	3	174 (47-448)	18	97 (63-144)	16	99 (62-151)

Supplementary Table S5A. Continued

			1980-1984		1985-1989		1990-1994		1995-1999		2000-2004		2005-2009		2010-2014		2015-2018		1980-2018		1985-2018	
**Cause of death (ICD9 code)**	**Area**	**Sex**	**Obs**	**SMR (90% CI)**	**Obs**	**SMR (CI 90%)**	**Obs**	**SMR (90% CI)**	**Obs**	**SMR (90% CI)**	**Obs**	**SMR (90% CI)**	**Obs**	**SMR (90% CI)**	**Obs**	**SMR (90% CI)**	**Obs**	**SMR (90% CI)**	**Obs**	**SMR (90% CI)**	**Obs**	**SMR (90% CI)**
Malignant neoplasms of lymphoid-haematopoietic and related tissue (200-208)	*Red area*	M	73	112 (92-137)	72	101 (82-123)	79	104 (85-125)	88	106 (88-127)	82	95 (79-115)	83	87 (72-104)	96	103 (87-122)	83	101 (84-121)	656	101 (94-107)	583	99 (93-106)
		F	50	95 (74-121)	56	88 (69-109)	79	105 (86-126)	65	81 (65-100)	85	101 (83-120)	71	85 (69-103)	74	84 (69-102)	65	89 (71-109)	545	91 (84-97)	495	90 (84-97)
	Red area A	M	33	113 (83-152)	28	88 (62-120)	35	103 (76-137)	38	104 (78-136)	40	105 (79-136)	40	93 (70-121)	42	99 (76-129)	33	88 (64-117)	289	99 (89-109)	256	97 (87-108)
		F	20	85 (56-123)	22	76 (51-108)	36	106 (79-140)	30	83 (60-113)	35	91 (67-120)	40	105 (79-136)	33	81 (60-109)	34	100 (74-133)	250	91 (82-101)	230	92 (82-102)
Diabetes mellitus (250)	*Red area*	M	67	91 (73-111)	73	101 (82-123)	52	75 (59-94)	72	103 (84-125)	57	79 (63-99)	77	94 (77-113)	96	104 (88-124)	100	121 (102-143)	594	97 (90-104)	527	97 (91-105)
		F	130	89 (77-103)	168	113 (99-129)	113	88 (75-103)	112	99 (84-116)	119	108 (92-126)	136	115 (99-132)	149	125 (108-143)	122	127 (108-147)	1049	107 (102-113)	919	110 (104-116)
	Red area A	M	27	82 (58-113)	38	117 (88-154)	28	91 (65-125)	23	75 (51-106)	28	88 (63-121)	32	88 (64-118)	52	125 (98-158)	51	135 (105-170)	279	102 (92-112)	252	104 (94-116)
		F	69	104 (84-127)	76	112 (91-135)	52	89 (69-112)	47	91 (70-116)	51	100 (78-127)	57	104 (83-130)	69	125 (101-152)	48	108 (84-138)	469	104 (96-113)	400	104 (96-113)
Diseases of the circulatory system (390-459)	*Red area*	M	1706	103 (99-107)	1534	107 (103-112)	1510	114 (109-119)	1422	106 (101-111)	1409	118 (112-123)	1309	117 (111-122)	1282	117 (112-123)	984	114 (108-120)	11156	111 (110-113)	9450	113 (111-115)
		F	1802	102 (98-106)	1781	109 (105-113)	1817	113 (109-118)	1834	113 (108-117)	1744	112 (108-117)	1613	108 (103-112)	1739	114 (110-119)	1427	117 (112-123)	13757	111 (109-112)	11955	112 (111-114)
	Red area A	M	775	103 (97-110)	727	113 (106-120)	724	123 (116-131)	656	112 (105-120)	663	126 (118-135)	625	126 (118-135)	610	123 (115-131)	473	120 (111-129)	5253	117 (115-120)	4478	120 (117-123)
		F	848	105 (99-111)	824	109 (103-116)	945	129 (122-136)	858	115 (109-122)	851	118 (111-125)	773	112 (105-118)	875	124 (117-131)	731	131 (123-139)	6705	117 (115-120)	5857	119 (117-122)
Heart diseases (390-429)	*Red area*	M	1080	98 (93-103)	926	99 (94-105)	996	110 (105-116)	967	101 (96-106)	1025	116 (111-123)	976	118 (112-125)	951	115 (109-122)	711	110 (103-117)	7632	108 (106-110)	6552	110 (108-112)
		F	1052	94 (89-99)	1010	103 (98-108)	1083	105 (100-111)	1215	109 (104-114)	1212	111 (105-116)	1126	105 (100-110)	1248	114 (109-119)	1030	116 (110-122)	8976	107 (105-109)	7924	109 (107-111)
	Red area A	M	490	98 (91-106)	436	104 (96-113)	457	114 (105-123)	428	102 (94-111)	473	123 (113-132)	467	128 (119-139)	453	121 (112-131)	330	111 (101-122)	3534	112 (109-115)	3044	114 (111-118)
		F	508	99 (92-107)	457	101 (93-109)	541	115 (107-124)	544	107 (99-115)	591	117 (109-125)	544	110 (102-118)	610	120 (112-129)	509	125 (116-134)	4304	111 (109-114)	3796	113 (110-116)
Ischaemic heart diseases (410-414)	*Red area*	M	607	98 (91-104)	543	103 (96-111)	574	112 (105-120)	561	108 (101-116)	570	121 (112-129)	559	121 (113-130)	523	121 (113-131)	353	114 (104-125)	4290	111 (109-114)	3683	114 (111-117)
		F	426	88 (81-95)	445	108 (100-117)	479	106 (98-114)	540	116 (108-125)	570	119 (111-127)	562	115 (107-123)	536	119 (110-127)	356	118 (108-129)	3914	111 (108-114)	3488	114 (111-118)
	Red area A	M	258	92 (83-102)	248	105 (95-117)	250	110 (99-122)	234	103 (92-115)	257	124 (111-137)	274	135 (122-149)	258	132 (119-146)	154	109 (95-124)	1933	113 (108-117)	1675	117 (112-121)
		F	200	91 (80-102)	190	101 (89-114)	244	118 (106-131)	239	113 (101-125)	276	125 (113-138)	281	124 (112-137)	266	127 (114-141)	180	130 (114-147)	1876	115 (111-120)	1676	119 (115-124)
Diseases of the digestive system (520-579)	*Red area*	M	283	103 (93-113)	266	119 (107-131)	216	116 (104-130)	178	116 (102-132)	160	110 (96-126)	146	109 (95-125)	166	132 (115-150)	119	115 (99-134)	1534	114 (109-119)	1251	117 (111-122)
		F	187	116 (103-131)	180	114 (100-128)	146	91 (79-105)	186	121 (107-137)	145	97 (85-112)	156	112 (98-128)	179	128 (113-145)	148	125 (108-143)	1327	113 (107-118)	1140	112 (107-118)
	Red area A	M	146	119 (103-136)	129	129 (111-149)	110	134 (113-157)	97	144 (121-170)	73	113 (92-137)	71	118 (96-144)	85	148 (122-177)	55	116 (92-145)	766	127 (120-135)	620	129 (121-138)
		F	103	142 (120-167)	97	134 (112-159)	70	96 (78-118)	96	138 (116-163)	73	107 (87-130)	59	92 (73-114)	81	125 (103-151)	66	121 (97-148)	645	120 (112-128)	542	116 (108-125)

Supplementary Table S5B. Sensitivity analysis. Observed number of deaths (Obs), standardised mortality ratios (SMR) and 90% confidence interval (CI) by cause of death, area, sex and calendar period. The *Red area* includes the 30 municipalities of the Veneto Region connected to the perfluoroalkyl and polyfluoroalkyl substances (PFAS)-contaminated aqueduct. The *Red area A* includes the subset of 13 municipalities with PFAS contamination of the groundwater too. The reference population is the population of the three provinces of Vicenza, Padua and Verona (northern Italy) minus the 30 municipalities of the Red Area. 1980-2018

			**1980-1984**		**1985-1989**		**1990-1994**		**1995-1999**		**2000-2004**		**2005-2009**		**2010-2014**		**2015-2018**		**1980-2018**		**1985-2018**	
**Cause of death (ICD9 code)**	**Area**	**Sex**	**Obs**	**SMR (90% CI)**	**Obs**	**SMR (90% CI)**	**Obs**	**SMR (90% CI)**	**Obs**	**SMR (90% CI)**	**Obs**	**SMR (90% CI)**	**Obs**	**SMR (90% CI)**	**Obs**	**SMR (90% CI)**	**Obs**	**SMR (90% CI)**	**Obs**	**SMR (90% CI)**	**Obs**	**SMR (90% CI)**
All causes (001-999)	*Red area*	M	4037	104 (101-107)	3957	108 (105-111)	3833	108 (105-111)	3729	107 (104-110)	3663	111 (108-114)	3539	108 (105-111)	3763	113 (110-117)	3108	114 (110-117)	29629	109 (108-110)	25592	110 (109-111)
		F	3489	104 (101-107)	3546	106 (103-109)	3580	107 (104-110)	3652	106 (103-109)	3627	105 (102-108)	3719	105 (102-108)	4211	111 (108-114)	3694	114 (111-117)	29518	107 (106-108)	26029	108 (107-109)
	Red area A	M	1839	105 (101-109)	1808	110 (106-114)	1781	113 (109-118)	1717	112 (107-116)	1641	113 (108-117)	1603	110 (105-114)	1732	115 (110-119)	1459	116 (111-122)	13580	111 (110-113)	11741	113 (111-114)
		F	1659	109 (104-113)	1615	106 (101-110)	1766	116 (111-120)	1717	109 (105-114)	1733	108 (104-113)	1746	107 (103-111)	2009	114 (110-119)	1773	119 (114-124)	14018	111 (109-112)	12359	111 (110-113)
Malignant neoplasms (140-208)	*Red area*	M	1075	101 (96-106)	1238	106 (101-111)	1265	106 (102-112)	1223	107 (102-112)	1220	110 (104-115)	1158	101 (96-106)	1221	110 (105-115)	930	109 (103-115)	9330	106 (104-108)	8255	107 (105-109)
		F	642	95 (89-101)	729	94 (88-100)	754	93 (87-98)	784	95 (89-100)	795	94 (88-99)	885	99 (94-105)	907	99 (94-104)	725	98 (92-104)	6221	96 (94-98)	5579	96 (94-98)
	Red area A	M	444	93 (86-101)	517	100 (92-107)	557	106 (99-114)	540	107 (100-115)	527	107 (99-114)	475	93 (86-100)	538	106 (99-114)	426	109 (100-118)	4024	102 (100-105)	3580	104 (101-107)
		F	287	94 (85-104)	301	86 (78-94)	353	96 (88-105)	370	99 (91-108)	371	96 (88-104)	411	101 (93-109)	411	97 (90-106)	330	96 (88-105)	2834	96 (93-99)	2547	96 (93-99)
Malignant neoplasm of liver and intrahepatic bile ducts (155)	*Red area*	M	58	125 (99-155)	66	105 (85-129)	83	106 (87-127)	94	123 (103-146)	86	111 (92-133)	80	100 (82-120)	93	117 (98-139)	64	107 (86-132)	624	111 (104-119)	566	110 (102-118)
		F	22	79 (53-108)	37	122 (91-156)	31	90 (65-118)	43	115 (88-145)	35	96 (71-124)	42	118 (90-149)	37	98 (73-126)	27	98 (69-131)	274	103 (93-113)	252	105 (95-117)
	Red area A	M	146	119 (103-136)	129	131 (112-151)	110	135 (115-158)	97	145 (122-172)	73	114 (93-138)	71	119 (97-145)	85	151 (125-181)	55	117 (92-147)	284	113 (102-125)	259	113 (101-125)
		F	103	144 (122-170)	97	136 (114-161)	70	96 (78-117)	96	140 (117-166)	73	107 (87-130)	59	92 (74-115)	81	127 (105-153)	66	122 (99-150)	118	97 (83-113)	112	103 (88-121)
Malignant neoplasm of liver (155.0)	*Red area*	M	37	125 (93-164)	34	96 (71-128)	53	98 (77-123)	45	97 (74-124)	38	93 (70-122)	34	100 (74-133)	51	111 (87-141)	29	118 (84-161)	321	103 (94-113)	284	101 (91-111)
		F	13	81 (48-128)	13	108 (64-172)	17	96 (61-144)	21	127 (85-183)	12	80 (46-130)	15	124 (77-192)	13	95 (56-152)	7	93 (44-175)	111	100 (85-118)	98	104 (87-123)
	Red area A	M	16	122 (76-185)	12	77 (44-124)	26	109 (76-151)	23	112 (77-159)	19	105 (69-154)	22	144 (98-206)	24	115 (79-161)	12	106 (61-171)	154	110 (97-127)	138	110 (95-127)
		F	4	55 (19-126)	4	73 (25-168)	6	75 (33-149)	13	176 (104-280)	5	74 (29-155)	8	146 (73-264)	7	112 (52-210)	<3	29 (1-136)	48	96 (74-122)	44	103 (79-132)
Malignant neoplasm of pancreas (157)	*Red area*	M	50	127 (99-161)	52	109 (85-137)	66	119 (96-146)	57	106 (84-132)	64	105 (84-129)	66	103 (83-127)	96	130 (109-154)	63	102 (82-126)	514	112 (104-121)	464	111 (103-120)
		F	27	80 (56-110)	44	98 (75-126)	39	72 (54-94)	44	76 (58-97)	64	95 (76-117)	67	90 (72-110)	84	105 (87-126)	63	92 (74-114)	432	90 (83-97)	405	90 (83-98)
	Red area A	M	21	120 (81-173)	23	108 (74-153)	28	114 (81-157)	25	106 (73-148)	30	110 (79-149)	29	101 (72-138)	45	134 (103-172)	26	92 (64-127)	227	111 (100-123)	206	110 (98-123)
		F	13	85 (50-136)	13	64 (38-101)	17	70 (44-105)	14	53 (32-84)	30	98 (70-133)	31	91 (66-122)	46	126 (97-161)	28	89 (63-121)	192	88 (77-99)	179	88 (77-99)
Malignant neoplasm of trachea, bronchus and lung (162)	*Red area*	M	339	98 (90-108)	402	109 (100-118)	461	125 (116-135)	366	105 (96-114)	359	112 (102-122)	341	111 (101-121)	304	110 (100-121)	235	115 (103-128)	2807	110 (107-114)	2468	112 (109-116)
		F	49	87 (67-108)	49	75 (58-93)	60	81 (64-99)	68	78 (63-94)	78	79 (65-94)	105	100 (85-117)	105	89 (75-103)	93	97 (81-115)	607	87 (81-93)	558	87 (81-93)
	Red area A	M	137	90 (78-104)	167	102 (90-116)	181	112 (98-126)	165	107 (94-122)	149	104 (91-120)	125	91 (78-105)	131	105 (90-122)	96	102 (86-121)	1151	102 (97-107)	1014	104 (98-109)
		F	25	99 (69-138)	23	78 (53-110)	24	72 (50-101)	36	92 (68-121)	30	67 (48-90)	50	105 (82-133)	50	92 (72-117)	43	97 (74-125)	281	88 (80-97)	256	87 (78-97)
Malignant neoplasm of testis (186)	*Red area*	M	<3	54 (2-259)	6 186 (81-368)						<3 0 (0-88)								7	91 (42-172)	6	103 (45-203)
	Red area A	M	<3	92 (4-436)	5 263 (103-552)						<3 0 (0-147)								6	132 (57-260)	5	144 (57-302)
Malignant neoplasm of ovary (183)	*Red area*	F	30	103 (74-140)	40	101 (76-131)	32	77 (56-103)	42	115 (87-149)	41	107 (81-139)	38	97 (72-127)	37	91 (68-120)	41	120 (91-155)	301	101 (91-111)	271	100 (91-111)
	Red area A	F	11	85 (48-140)	19	107 (70-157)	11	59 (33-98)	17	103 (66-155)	20	114 (76-166)	13	72 (43-115)	18	97 (62-143)	14	88 (53-137)	123	91 (78-105)	112	91 (77-107)
Malignant neoplasm of corpus uteri (182)	*Red area*	F	5	78 (31-164)	6	108 (47-214)	12	138 (79-223)	6	77 (33-151)	11	121 (68-200)	7	75 (35-140)	9	90 (47-158)	10	117 (63-198)	66	101 (81-124)	61	103 (83-128)
	Red area A	F	4	139 (47-318)	3	120 (32-310)	6	153 (67-303)	<3	57 (10-178)	5	121 (47-253)	4	93 (32-213)	3	65 (18-168)	<3	50 (9-158)	29	97 (70-132)	25	93 (64-129)
Malignant neoplasm of breast (174)	*Red area*	F	141	113 (97-129)	143	99 (86-114)	146	96 (84-111)	155	98 (85-111)	110	75 (64-88)	159	105 (92-120)	153	102 (89-116)	124	97 (83-113)	1131	98 (93-103)	990	96 (91-101)
	Red area A	F	64	114 (92-141)	62	96 (77-118)	69	101 (82-124)	80	112 (92-134)	44	66 (50-84)	74	106 (87-129)	70	101 (82-123)	69	117 (94-142)	532	101 (94-109)	468	100 (92-108)
Malignant neoplasm of the thyroid gland (193)	*Red area*	M	3	108 (29-278)	<3	31 (1-147)	<3	83 (14-262)	<3	40 (2-189)	4	165 (56-377)	<3	85 (15-267)	<3	30 (1-140)	3	172 (47-445)	17	82 (52-123)	14	78 (47-122)
		F	5	91 (36-192)	7	122 (57-228)	4	59 (20-135)	4	75 (25-171)	8	152 (76-275)	<3	47 (8-147)	<3	44 (8-137)	3	79 (21-205)	35	85 (63-113)	30	84 (60-114)
	Red area A	M	<3	81 (3-385)	<3	70 (3-333)	<3	189 (33-595)	<3	91 (4-430)	4	371 (126-849)	<3	95 (4-450)	<3	65 (3-309)	<3	125 (5-593)	12	130 (75-210)	11	137 (77-227)
		F	<3	81 (14-254)	3	115 (31-297)	3	99 (27-255)	3	125 (34-322)	3	125 (34-323)	<3	51 (2-242)	<3	0 (0-141)	3	171 (46-443)	18	96 (62-143)	16	98 (62-150)
Malignant neoplasm of kidney and other and unspecified urinary organs (189)	*Red area*	M	20	90 (60-131)	28	103 (73-141)	40	118 (89-153)	37	118 (88-155)	31	109 (79-148)	29	84 (60-115)	38	98 (73-128)	43	130 (100-168)	266	107 (96-118)	246	108 (97-120)
		F	12	92 (53-139)	16	108 (67-155)	22	109 (74-150)	15	90 (56-131)	18	102 (66-145)	17	83 (53-119)	24	116 (80-158)	22	124 (84-171)	146	104 (90-119)	134	105 (90-121)
	Red area A	M	7	71 (33-134)	11	91 (51-151)	17	113 (72-170)	10	72 (39-123)	12	95 (55-154)	7	45 (21-85)	15	85 (52-131)	27	178 (126-246)	106	95 (80-112)	99	97 (82-115)
		F	5	85 (33-179)	5	74 (29-156)	13	143 (85-227)	11	148 (83-244)	8	100 (50-181)	7	76 (35-142)	11	116 (65-192)	14	171 (104-268)	74	116 (94-140)	69	119 (96-145)

Supplementary Table S5B. Continued

			1980-1984		1985-1989		1990-1994		1995-1999		2000-2004		2005-2009		2010-2014		2015-2018		1980-2018		1985-2018	
**Cause of death (ICD9 code)**	**Area**	**Sex**	**Obs**	**SMR (90% CI)**	**Obs**	**SMR (90% CI)**	**Obs**	**SMR (90% CI)**	**Obs**	**SMR (90% CI)**	**Obs**	**SMR (90% CI)**	**Obs**	**SMR (90% CI)**	**Obs**	**SMR (90% CI)**	**Obs**	**SMR (90% CI)**	**Obs**	**SMR (90% CI)**	**Obs**	**SMR (90% CI)**
Malignant neoplasms of lymphoid, haematopoietic and related tissue (200-208)	*Red area*	M	73	113 (92-138)	72	101 (82-123)	79	104 (86-125)	88	107 (89-127)	82	95 (79-114)	83	86 (71-103)	96	104 (87-123)	83	101 (84-121)	656	101 (94-107)	583	99 (93-106)
		F	50	95 (74-118)	56	87 (69-107)	79	105 (86-125)	65	81 (65-98)	85	100 (83-119)	71	85 (69-102)	74	83 (68-100)	65	88 (71-107)	545	90 (84-97)	495	90 (83-97)
	Red area A	M	33	114 (84-153)	28	88 (62-121)	35	104 (77-138)	38	104 (78-137)	40	104 (79-136)	40	92 (70-120)	42	100 (76-129)	33	88 (64-117)	289	99 (89-109)	256	97 (87-108)
		F	20	84 (56-122)	22	76 (51-108)	36	106 (79-140)	30	83 (60-112)	35	90 (67-120)	40	104 (79-136)	33	81 (59-108)	34	100 (73-133)	250	91 (82-101)	230	92 (82-102)
Diabetes mellitus (250)	*Red area*	M	67	91 (73-111)	73	101 (82-123)	52	73 (58-93)	72	103 (84-125)	57	78 (62-97)	77	93 (77-113)	96	105 (88-124)	100	122 (103-144)	594	96 (90-103)	527	97 (90-105)
		F	130	89 (77-102)	168	114 (100-129)	113	87 (74-101)	112	98 (83-114)	119	108 (92-125)	136	115 (100-132)	149	125 (109-143)	122	128 (109-147)	1049	107 (102-113)	919	110 (104-117)
	Red area A	M	27	81 (57-112)	38	117 (88-154)	28	89 (63-122)	23	75 (51-107)	28	87 (62-120)	32	88 (64-118)	52	125 (98-158)	51	137 (107-172)	279	101 (92-112)	252	104 (94-116)
		F	69	104 (84-127)	76	113 (92-136)	52	88 (69-111)	47	90 (70-115)	51	101 (79-127)	57	105 (83-131)	69	126 (102-154)	48	109 (85-139)	469	104 (97-113)	400	105 (96-114)
Diseases of the circulatory system (390-459)	*Red area*	M	1706	103 (99-107)	1534	108 (103-113)	1510	115 (110-120)	1422	106 (102-111)	1409	119 (114-124)	1309	118 (112-123)	1282	119 (113-124)	984	115 (109-121)	11156	112 (110-114)	9450	114 (112-116)
		F	1802	102 (98-106)	1781	110 (105-114)	1817	114 (110-119)	1834	114 (109-118)	1744	113 (109-117)	1613	108 (104-113)	1739	115 (111-120)	1427	119 (114-124)	13757	111 (110-113)	11955	113 (111-115)
	Red area A	M	775	104 (97-110)	727	113 (107-121)	724	124 (117-132)	656	113 (105-120)	663	128 (120-136)	625	128 (119-136)	610	124 (116-133)	473	121 (112-130)	5253	118 (115-121)	4478	121 (118-124)
		F	848	105 (99-111)	824	110 (104-116)	945	130 (123-137)	858	116 (110-123)	851	119 (112-126)	773	112 (106-119)	875	125 (118-133)	731	132 (124-141)	6705	118 (116-121)	5857	120 (118-123)
Heart diseases (390-429)	*Red area*	M	1080	98 (93-103)	926	99 (94-105)	996	111 (105-117)	967	101 (96-106)	1025	118 (112-124)	976	120 (113-126)	951	117 (110-123)	711	110 (104-117)	7632	108 (106-111)	6552	110 (108-113)
		F	1052	94 (89-98)	1010	103 (98-109)	1083	106 (101-111)	1215	110 (104-115)	1212	111 (106-117)	1126	106 (100-111)	1248	115 (110-120)	1030	117 (111-123)	8976	107 (106-109)	7924	110 (108-112)
	Red area A	M	490	98 (91-106)	436	104 (96-113)	457	115 (106-124)	428	102 (94-111)	473	124 (115-134)	467	130 (120-140)	453	122 (113-132)	330	112 (102-122)	3534	112 (109-116)	3044	115 (112-119)
		F	508	99 (92-107)	457	101 (94-109)	541	116 (108-124)	544	107 (100-115)	591	117 (109-126)	544	110 (102-118)	610	121 (113-130)	509	126 (117-136)	4304	112 (109-115)	3796	114 (111-117)
Ischaemic heart diseases (410-414)	*Red area*	M	607	97 (91-104)	543	103 (96-111)	574	113 (105-121)	561	109 (101-116)	570	122 (114-131)	559	123 (114-132)	523	123 (114-132)	353	115 (105-126)	4290	112 (109-115)	3683	115 (112-118)
		F	426	88 (81-95)	445	109 (101-117)	479	106 (99-115)	540	117 (109-126)	570	120 (112-129)	562	116 (108-124)	536	120 (111-129)	356	119 (109-130)	3914	112 (109-115)	3488	115 (112-119)
	Red area A	M	258	92 (83-102)	248	105 (95-117)	250	111 (100-123)	234	104 (93-115)	257	125 (113-139)	274	136 (123-151)	258	134 (120-148)	154	110 (96-125)	1933	113 (109-118)	1675	118 (113-122)
		F	200	90 (80-101)	190	101 (89-114)	244	119 (107-132)	239	114 (102-127)	276	126 (114-139)	281	125 (113-138)	266	128 (116-142)	180	131 (116-149)	1876	116 (112-121)	1676	121 (116-125)
Diseases of the digestive system (520-579)	*Red area*	M	283	103 (93-113)	266	120 (108-133)	216	118 (105-132)	178	118 (103-133)	160	111 (97-126)	146	110 (95-126)	166	134 (118-153)	119	117 (100-136)	1534	115 (110-120)	1251	118 (113-124)
		F	187	118 (104-133)	180	115 (101-129)	146	91 (79-104)	186	123 (108-138)	145	98 (85-111)	156	113 (98-128)	179	130 (114-146)	148	126 (110-144)	1327	114 (108-119)	1140	113 (107-118)
	Red area A	M	146	119 (103-136)	129	131 (112-151)	110	135 (115-158)	97	145 (122-172)	73	114 (93-138)	71	119 (97-145)	85	151 (125-181)	55	117 (92-147)	766	128 (121-136)	620	131 (122-140)
		F	103	144 (122-170)	97	136 (114-161)	70	96 (78-117)	96	140 (117-166)	73	107 (87-130)	59	92 (74-115)	81	127 (105-153)	66	122 (99-150)	645	121 (113-129)	542	117 (109-126)

Supplementary Table S6. Observed number of deaths (Obs) and standardised mortality ratio (SMR) in three broad calendar periods, ratio of SMR 1980-1989 to SMR 1980-2018, ratio of SMR 2010-2018 to SMR 1980-2018, and exact one-sided mid-p-values, by causes of death, area and sex. *Red area* includes the 30 municipalities connected to the perfluoroalkyl and polyfluoroalkyl substances (PFAS)-contaminated aqueduct. The *Red area A* includes the subset of 13 municipalities with PFAS contamination of the groundwater too

Area	Sex	Obs 1980-2018	SMR 1980-2018	Obs 1980-1989	SMR 1980-1989	Obs 2010-2018	SMR 2010-2018	RR 1980-1989 to 1980-2018 (mid-p-value)	RR 2010-2018 to 1980-2018 (mid-p-value)	Cause of death (ICD-9 code)
Red area	M	29,629	108	7994	106	6871	113	0.98 (0.05)	1.05 (<0.01)	All causes (001-999)
	F	29,518	107	7035	105	7905	111	0.98 (0.06)	1.04 (<0.01)	
Red area A	M	13,580	111	3647	107	3191	115	0.96 (0.01)	1.04 (0.02)	
	F	14,018	110	3274	106	3782	116	0.96 (0.02)	1.05 (<0.01)	
Red area	M	9330	106	2313	103	2151	109	0.97 (0.08)	1.03 (0.10)	Malignant neoplasms (140-208)
	F	6221	96	1371	94	1632	99	0.98 (0.22)	1.03 (0.11)	
Red area A	M	4024	102	961	96	964	107	0.94 (0.03)	1.05 (0.07)	
	F	2834	96	588	90	741	97	0.94 (0.06)	1.01 (0.39)	
Red area	M	624	110	124	113	157	112	1.03 (0.62)	1.02 (0.41)	Malignant neoplasm of liver and intrahepatic bile ducts (155)
	F	274	102	59	101	64	98	0.99 (0.48)	0.96 (0.62)	
Red area A	M	284	113	54	110	69	108	0.97 (0.43)	0.96 (0.64)	
	F	118	97	22	83	18	61	0.86 (0.24)	0.63 (0.98)	
Red area	M	321	103	71	108	80	113	1.05 (0.66)	1.10 (0.20)	Malignant neoplasm of liver (155.0)
	F	111	100	26	93	20	95	0.93 (0.37)	0.95 (0.58)	
Red area A	M	154	111	28	97	36	111	0.87 (0.24)	1.00 (0.49)	
	F	48	96	8	63	8	82	0.66 (0.11)	0.85 (0.65)	
Red area	M	514	112	102	116	159	116	1.04 (0.66)	1.04 (0.31)	Malignant neoplasm of pancreas (157)
	F	432	90	71	78	147	99	0.87 (0.10)	1.10 (0.12)	
Red area A	M	227	110	44	113	71	113	1.03 (0.59)	1.03 (0.39)	
	F	192	88	26	73	74	109	0.83 (0.17)	1.24 (0.04)	
Red area	M	2807	110	741	103	539	112	0.94 (0.04)	1.02 (0.34)	Malignant neoplasm of trachea, bronchus and lung (162)
	F	607	87	98	81	198	93	0.93 (0.24)	1.07 (0.17)	
Red area A	M	1151	101	304	96	227	103	0.95 (0.19)	1.02 (0.38)	
	F	281	89	48	88	93	95	0.99 (0.48)	1.07 (0.26)	
Red area	F	1131	98	284	105	277	100	1.07 (0.88)	1.02 (0.37)	Malignant neoplasm of breast (174)
Red area A	F	532	101	126	104	139	108	1.03 (0.63)	1.07 (0.21)	
Red area	F	66	101	11	92	19	103	0.91 (0.40)	1.02 (0.45)	Malignant neoplasm of corpus uteri (182)
Red area A	F	29	97	7	131	5	58	1.35 (0.79)	0.60 (0.88)	
Red area	F	301	101	70	102	78	104	1.01 (0.54)	1.03 (0.39)	Malignant neoplasm of ovary (183)
Red area A	F	123	91	30	98	32	92	1.08 (0.67)	1.01 (0.46)	
Red area	M	7	71	5	0	<3	0			Malignant neoplasm of testis (186)
Red area A	M	6	131	3	0	<3	0			

Supplementary Table S6. Continued

**Area**	**Sex**	**Obs 1980-2018**	**SMR 1980-2018**	**Obs 1980-1989**	**SMR 1980-1989**	**Obs 2010-2018**	**SMR 2010-2018**	**RR 1980-1989 to 1980-2018 (mid-p-value)**	**RR 2010-2018 to 1980-2018 (mid-p-value)**	**Cause of death (ICD-9 code)**
Red area	M	266	106	48	98	81	112	0.92 (0.30)	1.06 (0.31)	Malignant neoplasm of kidney and other and unspecified urinary organs (189)
	F	146	104	28	102	46	119	0.98 (0.47)	1.14 (0.18)	
Red area A	M	106	95	18	83	42	127	0.87 (0.29)	1.34 (0.03)	
	F	74	116	10	80	25	140	0.69 (0.12)	1.21 (0.17)	
Red area	M	17	83	4	69	4	76	0.83 (0.38)	0.92 (0.54)	Malignant neoplasm of the thyroid gland (193)
	F	35	86	12	106	5	61	1.23 (0.77)	0.71 (0.77)	
Red area A	M	12	131	<3	77	<3	87	0.59 (0.24)	0.66 (0.69)	
	F	18	97	5	98	3	103	1.01 (0.54)	1.06 (0.43)	
Red area	M	656	101	145	106	179	102	1.05 (0.72)	1.01 (0.44)	Malignant neoplasms of lymphoid, haematopoietic and related tissue (200-208)
	F	545	91	106	91	139	86	1.00 (0.51)	0.95 (0.74)	
Red area A	M	289	99	61	100	75	94	1.01 (0.54)	0.95 (0.67)	
	F	250	91	42	80	67	90	0.88 (0.20)	0.99 (0.53)	
Red area	M	594	97	140	96	196	112	0.99 (0.46)	1.15 (0.02)	Diabetes mellitus (250)
	F	1049	107	298	101	271	126	0.94 (0.16)	1.18 (<0.01)	
Red area A	M	279	102	65	99	103	130	0.97 (0.41)	1.27 (0.01)	
	F	469	104	145	108	117	117	1.04 (0.68)	1.13 (0.10)	
Red area	M	11,156	111	3240	105	2266	116	0.95 (<0.01)	1.05 (0.02)	Diseases of the circulatory system (390-459)
	F	13,757	111	3583	105	3166	115	0.95 (<0.01)	1.04 (0.02)	
Red area A	M	5253	117	1502	108	1083	122	0.92 (<0.01)	1.04 (0.08)	
	F	6705	117	1672	107	1606	127	0.91 (<0.01)	1.09 (<0.01)	
Red area	M	7632	108	2006	98	1662	113	0.91 (<0.01)	1.05 (0.03)	Heart diseases (390-429)
	F	8976	107	2062	98	2278	115	0.92 (<0.01)	1.07 (<0.01)	
Red area A	M	3534	112	926	101	783	117	0.90 (<0.01)	1.04 (0.11)	
	F	4304	111	965	100	1119	122	0.90 (<0.01)	1.1 (<0.01)	
Red area	M	4290	111	1150	100	876	118	0.90 (<0.01)	1.06 (0.04)	Ischaemic heart diseases (410-414)
	F	3914	111	871	97	892	119	0.87 (<0.01)	1.07 (0.02)	
Red area A	M	1933	113	506	98	412	122	0.87 (<0.01)	1.08 (0.06)	
	F	1876	115	390	96	446	128	0.83 (<0.01)	1.11 (0.01)	
Red area	M	1534	114	549	110	285	124	0.96 (0.20)	1.09 (0.08)	Diseases of the digestive system (520-579)
	F	1327	113	367	115	327	127	1.02 (0.63)	1.12 (0.02)	
Red area A	M	766	127	275	123	140	134	0.97 (0.30)	1.06 (0.26)	
	F	645	120	200	138	147	123	1.15 (0.97)	1.02 (0.38)	

Abbreviation: *ICD-9* International classification of diseases, 9th revision; *RR* Relative risk

Note: One-sided mid-p-values of the null hypothesis: H_0: RR 1980-1989 to 1980-2018 = 1, H_1: 1980-1989 to 1980-2018 <1 and H_0: RR 2010-2018 to 1980-2018 = 1, H_1: RR 2010-2018 to 1980-2018>1

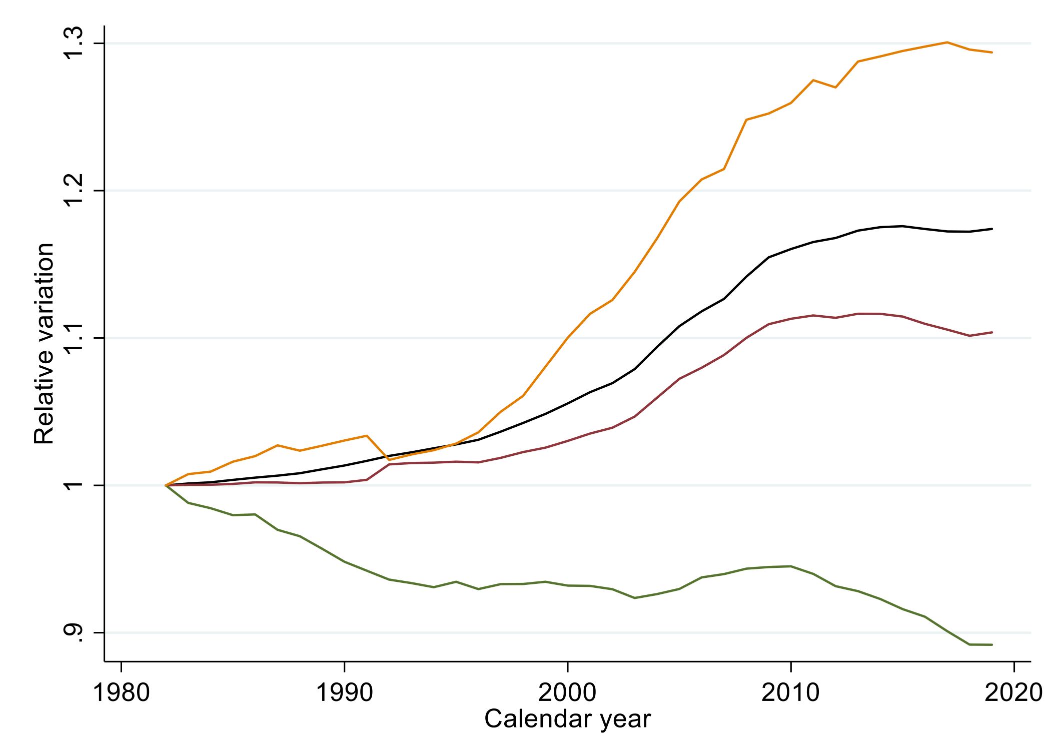

Supplementary Fig. S1. Relative variation of the size of the population. The reference calendar year is 1982. The black line indicates the provinces of Vicenza, Padua and Verona (northern Italy) as a whole. The red line indicates the 30 municipalities of the perfluoroalkyl and polyfluoroalkyl substances (PFAS)-contaminated *Red area*. The yellow line indicates the municipality of Lonigo. The green line indicates the municipality of Montagnana. 1982-2019. Source: Italian National Institute of Statistics (https://demo.istat.it/. Accessed 14 Feb 2024)

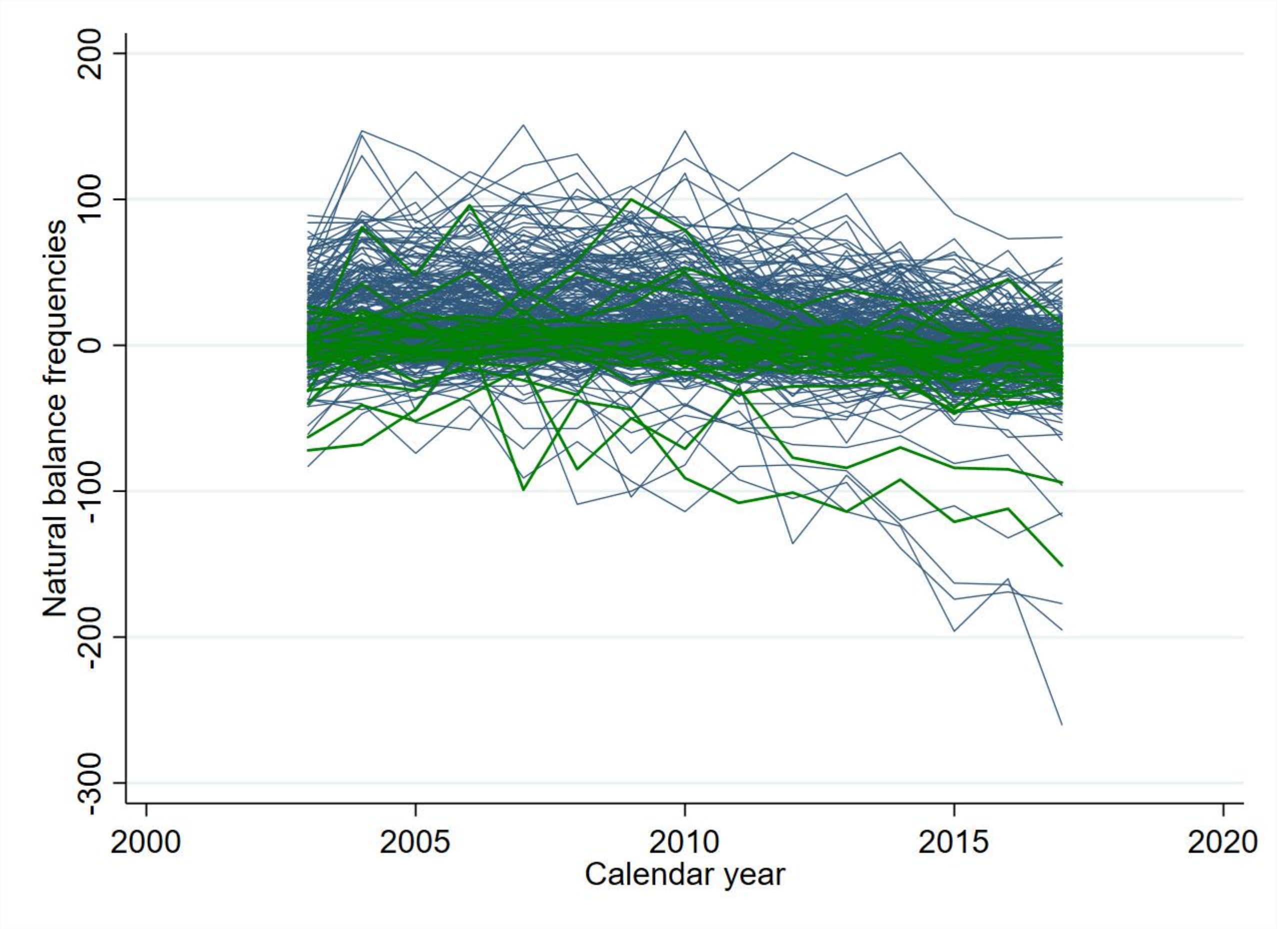

Supplementary Fig. S2. Natural balance of the population of the 321 municipalities of the provinces of Vicenza, Padua and Verona (northern Italy) as a whole by calendar year. The green lines indicate the 30 municipalities of the perfluoroalkyl and polyfluoroalkyl substances (PFAS)-contaminated *Red area*. The three province capital cities were excluded. Legnago and Montagnana were the municipalities of the *Red area* with the lowest natural balance. Lonigo had a positive natural balance. 2003-2017. Source: Italian National Institute of Statistics (https://demo.istat.it/. Accessed 14 Feb 2024)

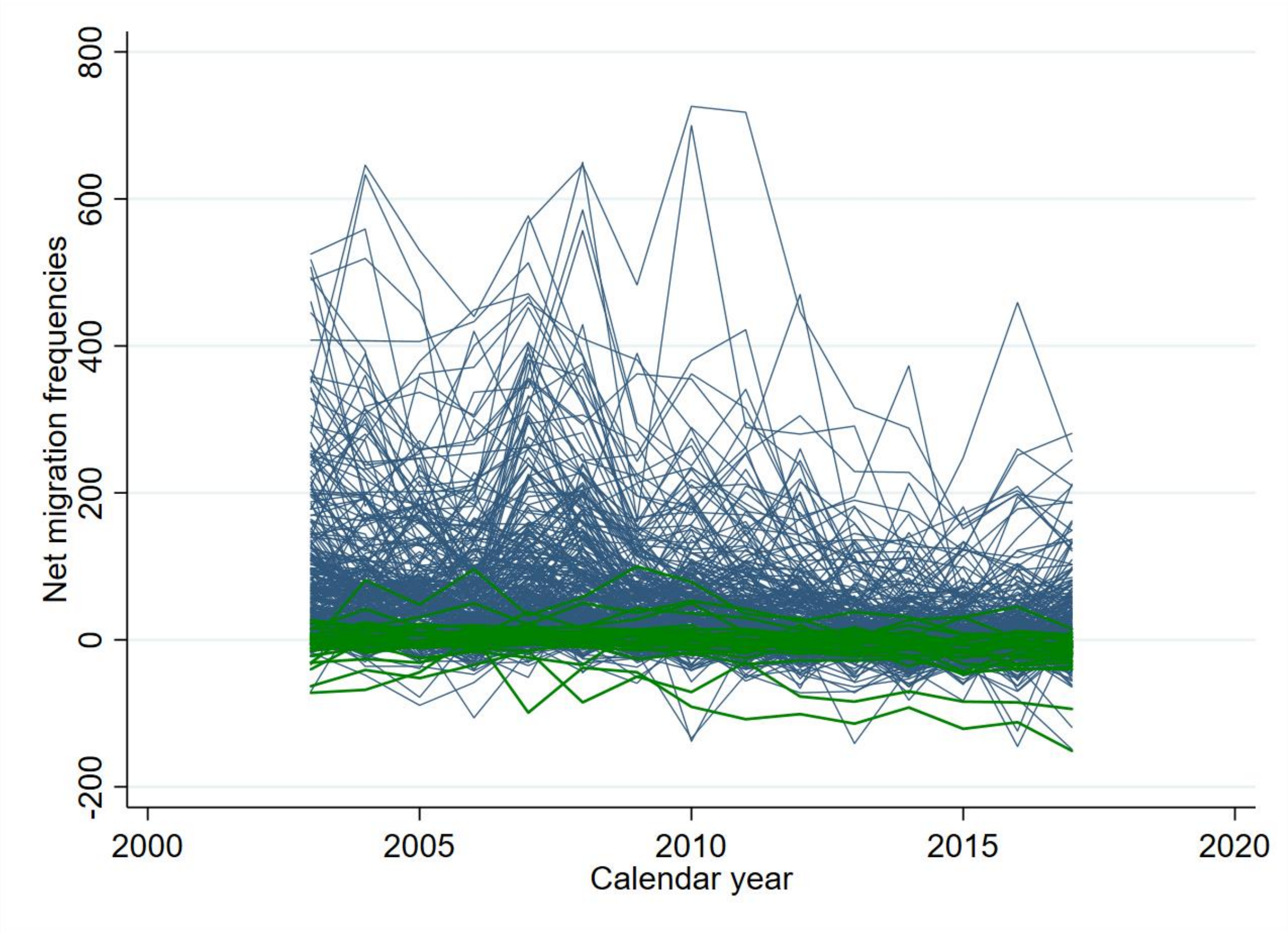

Supplementary Fig. S3. Net migration from the 321 municipalities of the provinces of Vicenza, Padua and Verona (northern Italy) as a whole by calendar year. The green lines indicate the 30 municipalities of the perfluoroalkyl and polyfluoroalkyl substances (PFAS)-contaminated *Red area*. The three province capital cities were excluded. Lonigo was the municipality of the *Red area* with the highest net migration. 2003-2017. Source: Italian National Institute of Statistics (https://demo.istat.it. Accessed 14 Feb 2024)

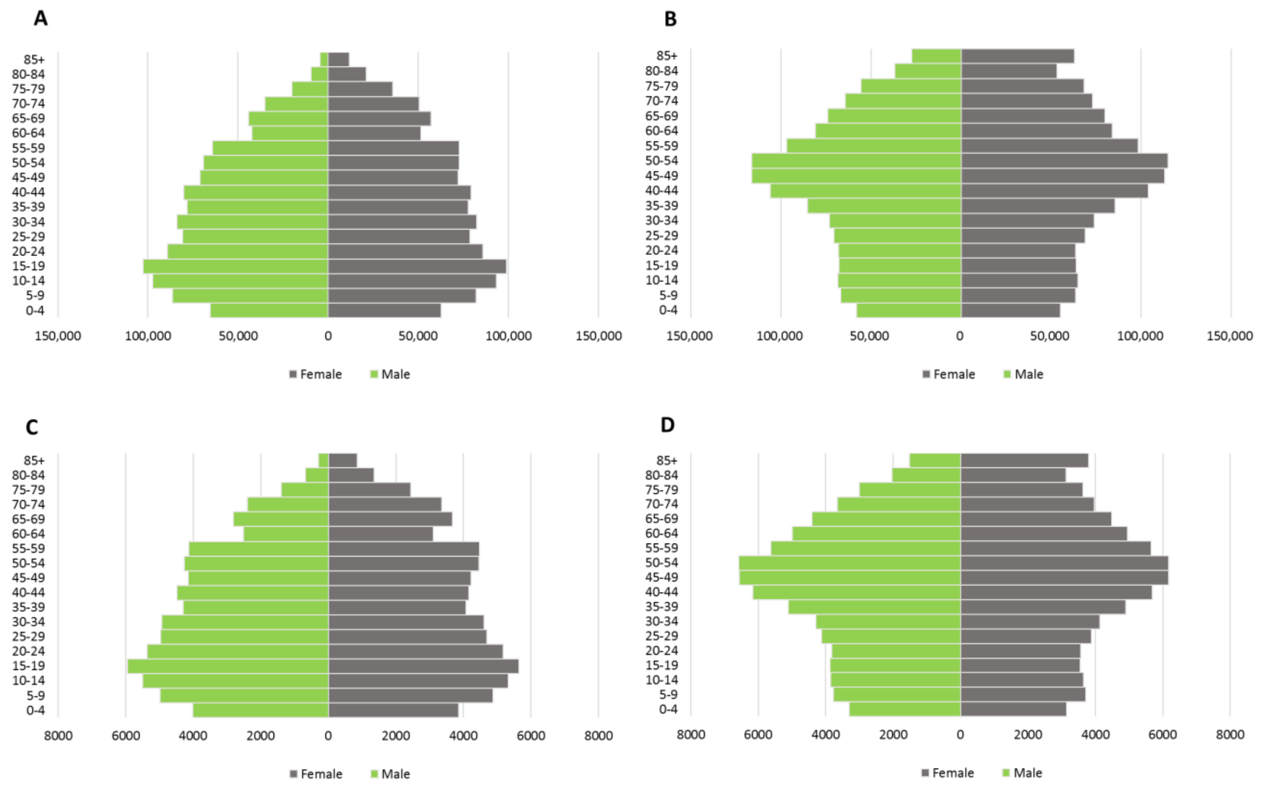

Supplementary Fig. S4. Age-sex pyramid of the population of the provinces of Vicenza, Padua and Verona (northern Italy) as a whole (panels A and B) and of the 30 municipalities of the perfluoroalkyl and polyfluoroalkyl substances (PFAS)-contaminated *Red area* (panels C and D). 1 January 1982 (panels A and C) and 1 January 2018 (panels B and D). Source: Italian National Institute of Statistics (https://demo.istat.it/. Accessed 14 Feb 2024)

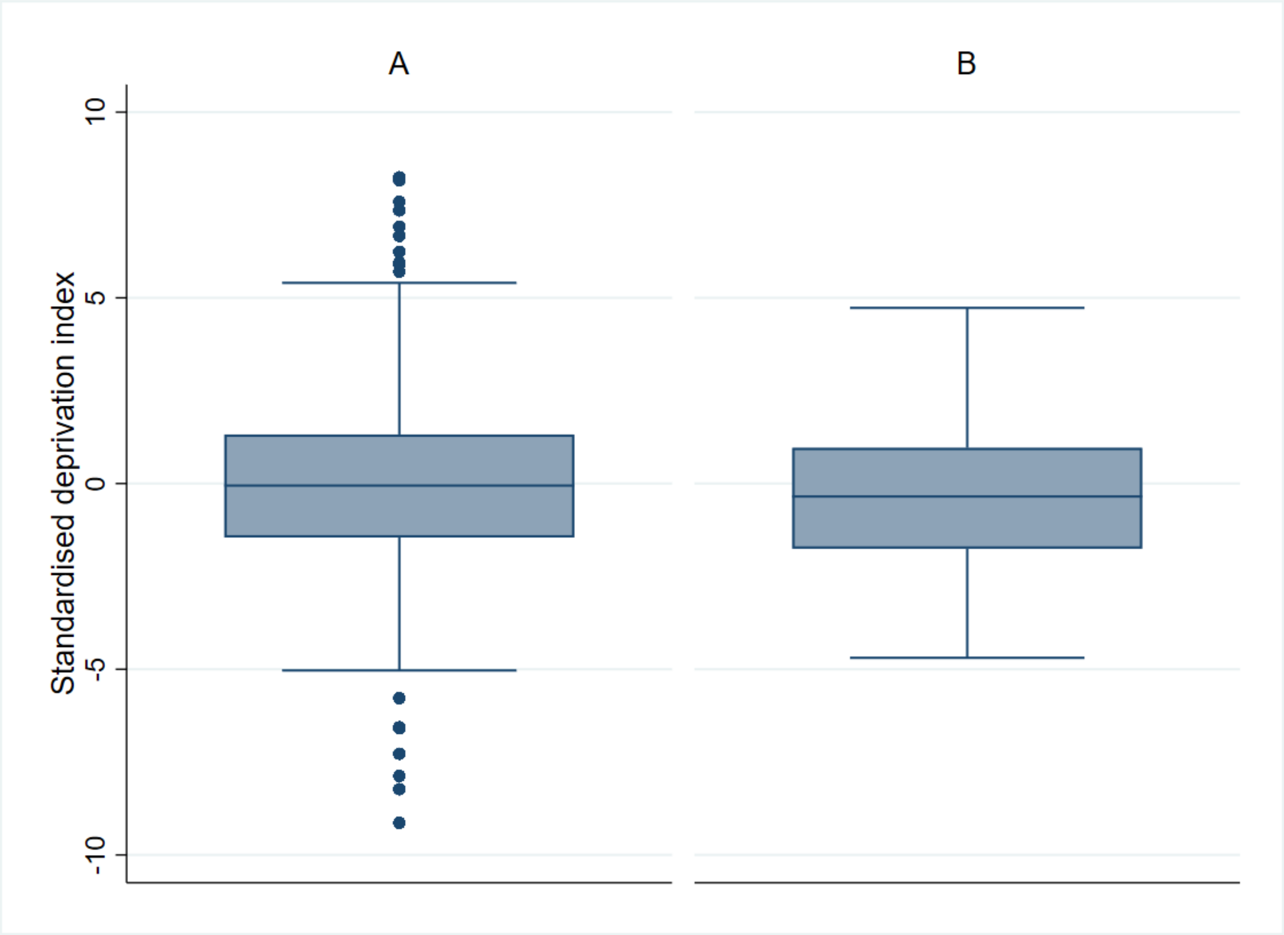

Supplementary Fig. S5. Box and whiskers plots of the standardised deprivation index in the provinces of Vicenza, Padua and Verona (northern Italy) as a whole (panel A) and in the 30 municipalities of the perfluoroalkyl and polyfluoroalkyl substances (PFAS)-contaminated *Red area* (panel B). 2011 census. Source: Rosano A, Pacelli B, Zengarini N, Costa G, Cislaghi C, Caranci N. Aggiornamento e revisione dell'indice di deprivazione italiano 2011 a livello di sezione di censimento [Update and review of the 2011 Italian deprivation index calculated at the census section level]. Epidemiol Prev. 2020;44:162-70

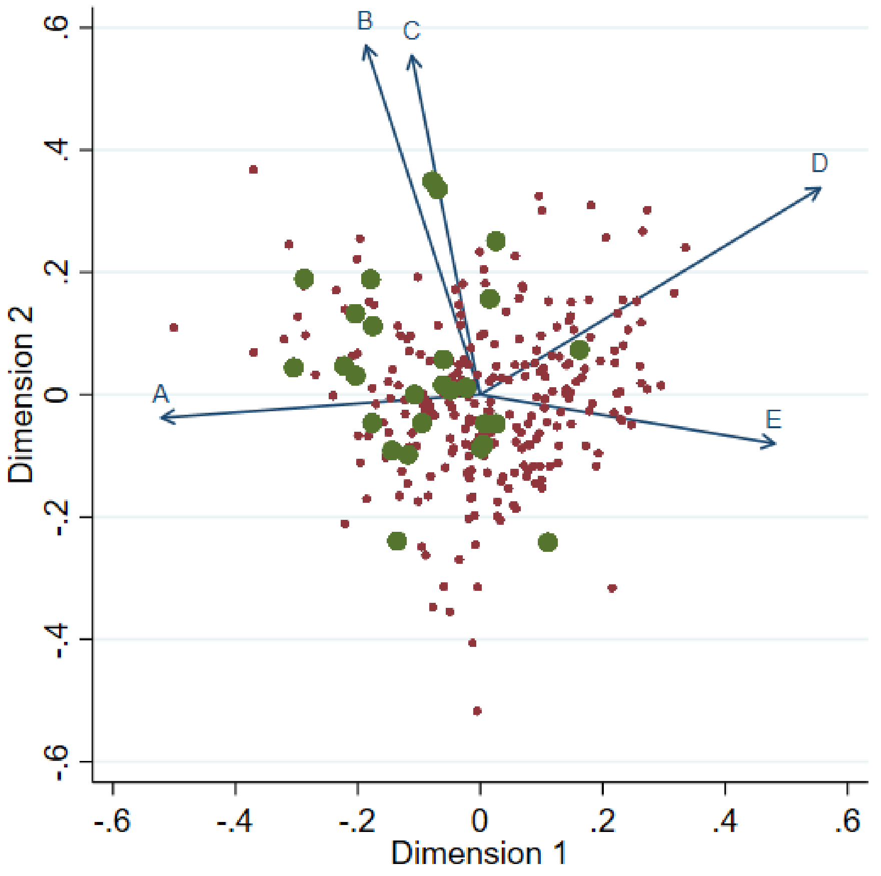

Supplementary Fig. S6. Biplot of the first two principal components of the five socio-demographic indicators of deprivation (low level of education (A), single parenting (B), unemployment (C), rental housing (D), and house crowding (E)). The red points indicate the 321 municipalities in the provinces of Vicenza, Padua and Verona (northern Italy) as a whole. The green points indicate the 30 municipalities of the perfluoroalkyl and polyfluoroalkyl substances (PFAS)-contaminated *Red area.* The arrows denote the vectors of the five indicators. 2011 census. Source: Rosano A, Pacelli B, Zengarini N, Costa G, Cislaghi C, Caranci N. Aggiornamento e revisione dell'indice di deprivazione italiano 2011 a livello di sezione di censimento [Update and review of the 2011 Italian deprivation index calculated at the census section level]. Epidemiol Prev. 2020;44:162-70

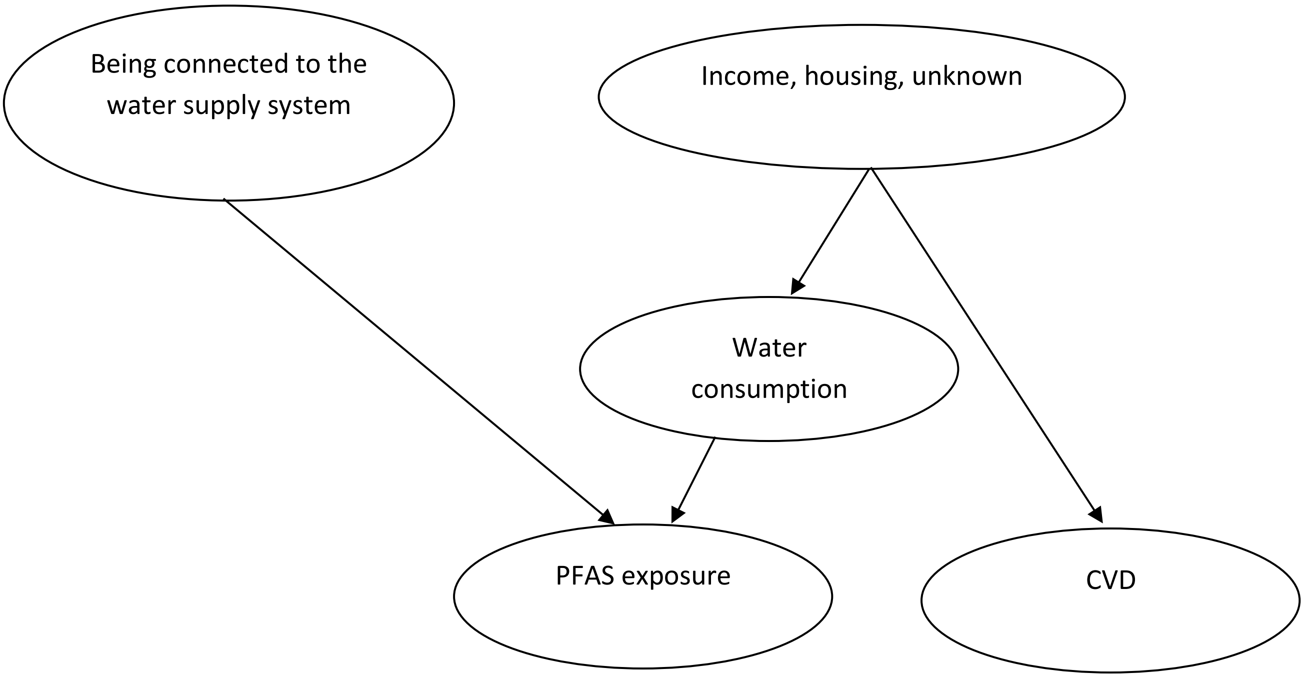

Supplementary Fig. S7. Directed acyclic graph of the assumed causal model. Abbreviations: *PFAS* per- and polyfluoroalkyl substances; *CVD* cardiovascular disease

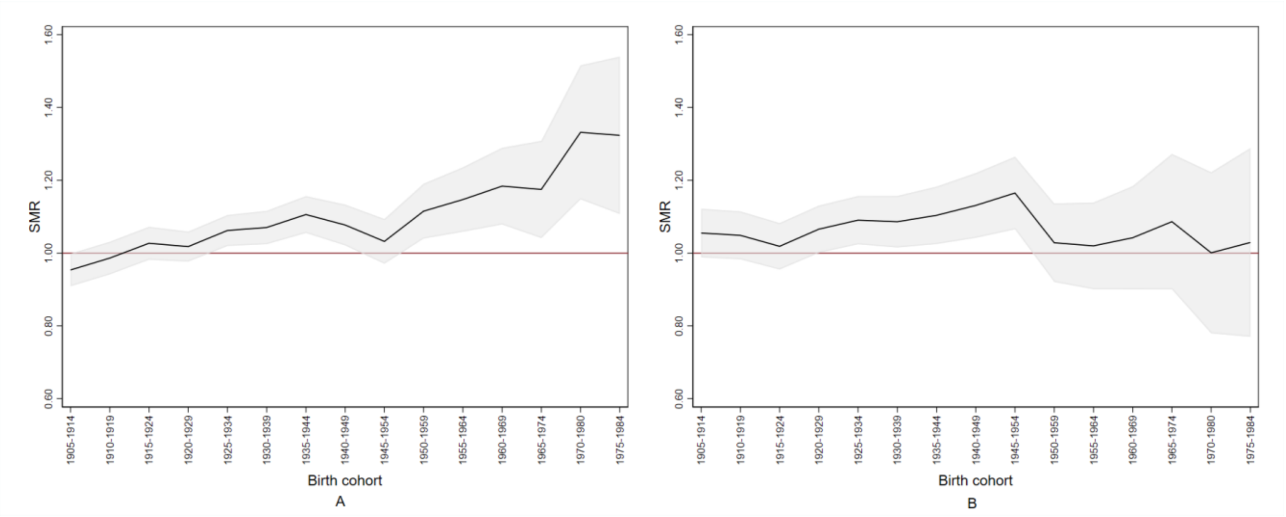

Supplementary Fig. S8. Sensitivity analysis. Cumulative standardised mortality ratio (SMR) for all causes by sex and birth cohort (1905-1984) in the perfluoroalkyl and polyfluoroalkyl substances (PFAS)-contaminated *Red area.* Panel A, males; panel B, females. The grey area indicates the 90% confidence band. The red line indicates the reference value. The reference population is the population of the three provinces of Vicenza, Padua and Verona (northern Italy) minus the 30 municipalities of the Red Area

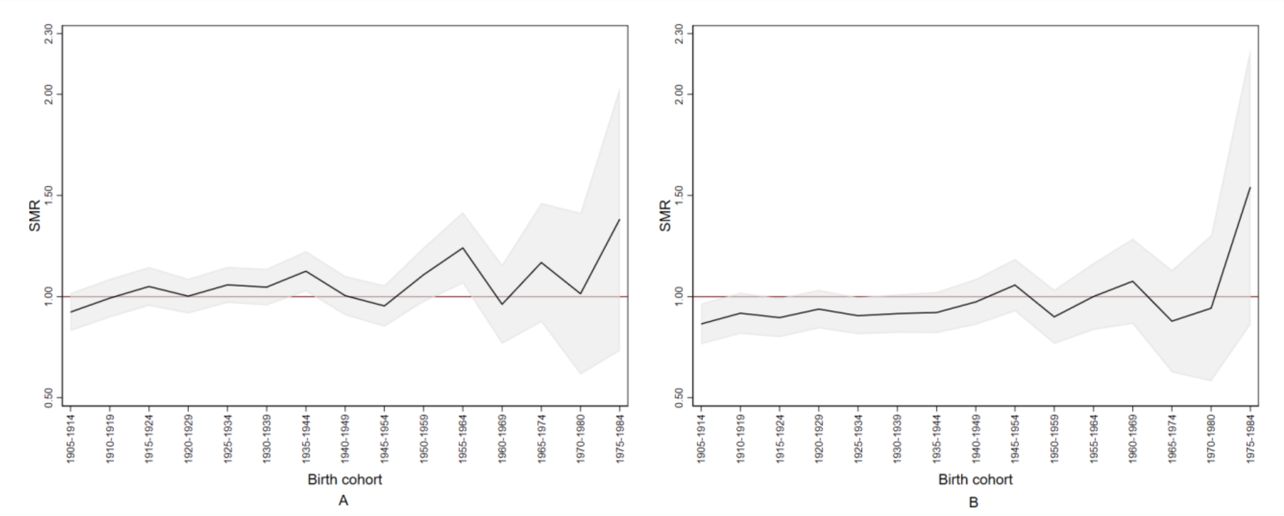

Supplementary Fig. S9. Sensitivity analysis. Cumulative standardised mortality ratio (SMR) for malignant neoplasms by sex and birth cohort (1905-1984) in the perfluoroalkyl and polyfluoroalkyl substances (PFAS)-contaminated *Red area.* Panel A, males; panel B, females. The grey area indicates the 90% confidence band. The red line indicates the reference value. The reference population is the population of the three provinces of Vicenza, Padua and Verona (northern Italy) minus the 30 municipalities of the Red Area
